# Arousal and Locomotion Differently Modulate Activity of Somatostatin Neurons across Cortex

**DOI:** 10.1523/ENEURO.0136-23.2023

**Published:** 2023-05-24

**Authors:** Christine F. Khoury, Noelle G. Fala, Caroline A. Runyan

**Affiliations:** 1 Center for the Neural Basis of Cognition, University of Pittsburgh, Pittsburgh, Pennsylvania 15213; 2 Department of Neuroscience, University of Pittsburgh, Pittsburgh, Pennsylvania 15260

**Keywords:** arousal, auditory cortex, inhibition, posterior parietal cortex, somatostatin

## Abstract

Arousal powerfully influences cortical activity, in part by modulating local inhibitory circuits. Somatostatin (SOM)-expressing inhibitory interneurons are particularly well situated to shape local population activity in response to shifts in arousal, yet the relationship between arousal state and SOM activity has not been characterized outside of sensory cortex. To determine whether SOM activity is similarly modulated by behavioral state across different levels of the cortical processing hierarchy, we compared the behavioral modulation of SOM-expressing neurons in auditory cortex (AC), a primary sensory region, and posterior parietal cortex (PPC), an association-level region of cortex, in mice. Behavioral state modulated activity differently in AC and PPC. In PPC, transitions to high arousal were accompanied by large increases in activity across the full PPC neural population, especially in SOM neurons. In AC, arousal transitions led to more subtle changes in overall activity, as individual SOM and Non-SOM neurons could be either positively or negatively modulated during transitions to high arousal states. The coding of sensory information in population activity was enhanced during periods of high arousal in AC, but not in PPC. Our findings suggest unique relationships between activity in local circuits and arousal across cortex, which may be tailored to the roles of specific cortical regions in sensory processing or the control of behavior.

## Significance Statement

The effects of arousal on brain networks are profound but vary across regions. Somatostatin (SOM) neurons may carry out some of the effects of arousal on local network activity in sensory cortex, by modulating response gain and decorrelating population activity. However, SOM neurons have not been well studied outside of sensory cortex, and so it is unknown whether SOM neurons are similarly affected by shifts in brain state throughout the cortex. Here, we have revealed specialization in the relationship between arousal and activity in SOM neurons that could contribute to the diversity of arousal-related impacts on local computation across cortical regions.

## Introduction

Arousal profoundly impacts brain activity, at both local and global scales (Livingstone and Hubel, 1981; Marrocco et al., 1994; Cantero et al., 1999; Gould et al., 2011; Grent-’t-Jong et al., 2011; Stringer et al., 2019), and affects sensory perception by tailoring sensory processing to match behavioral demands (Pinto et al., 2013; McGinley et al., 2015; Dadarlat and Stryker, 2017; Kuchibhotla et al., 2017). In sensory cortex, the response gain and signal-to-noise ratio of responses to sensory stimuli of individual neurons are enhanced with arousal (Niell and Stryker, 2010; Bennett et al., 2013; Polack et al., 2013; Fu et al., 2014; McGinley et al., 2015; Vinck et al., 2015; Mineault et al., 2016). At the population level, activity across neurons becomes decorrelated (Poulet and Petersen, 2008; Zhou et al., 2014; Vinck et al., 2015; Lin et al., 2019), which can enhance the encoding of sensory information by reducing redundancy and correlated noise (Averbeck et al., 2006).

In sensory cortex, these effects can be triggered by the release of neuromodulators linked to arousal, such as norepinephrine and acetylcholine, which act in part through inhibitory interneurons to influence local activity patterns (Fu et al., 2014; Chen et al., 2015; Kuchibhotla et al., 2017; Garcia-Junco-Clemente et al., 2019). The inhibitory cell class can be divided into three nonoverlapping subtypes, which express parvalbumin (PV), somatostatin (SOM), or vasoactive intestinal peptide (VIP), and exhibit distinct local connectivity patterns (Tremblay et al., 2016). SOM neurons are particularly sensitive to arousal state fluctuations, through direct activation of cholinergic and noradrenergic receptors (Kawaguchi and Shindou, 1998; Xiang et al., 1998; Beierlein et al., 2000; Fanselow et al., 2008; Chen et al., 2015; Kuchibhotla et al., 2017) and inhibition by VIP neurons (Pfeffer et al., 2013; Pi et al., 2013; Fu et al., 2014; Karnani et al., 2016; Dipoppa et al., 2018). In turn, SOM neurons can powerfully influence local network activity, as they densely innervate the local excitatory population (Fino and Yuste, 2011; Pfeffer et al., 2013; Xu et al., 2013). When they are active, SOM neurons enhance stimulus selectivity and response reliability (Adesnik et al., 2012; Wilson et al., 2012; Rikhye et al., 2021), control local population activity dynamics (Chen et al., 2015; Veit et al., 2017), and flexibly modulate excitatory responses to stimuli, based on behavioral relevance (Kato et al., 2015; Wang and Yang, 2018).

The impacts of arousal on sensory perception, and on coding in sensory cortex, have been well studied (Fu et al., 2014; Zhou et al., 2014; McGinley et al., 2015; Vinck et al., 2015; Pakan et al., 2016). It is unclear though how specialized or generalized the relationship between arousal and local inhibitory circuit function is across the cortical hierarchy. For example, while arousal-mediated and attention-mediated reductions in shared variability seem to improve sensory coding (Cohen and Maunsell, 2009; Goard and Dan, 2009), similar effects could be detrimental to the readout of perceptual decisions to control behavior in higher cortex (Runyan et al., 2017; Valente et al., 2021). The basic structure of local circuits is highly conserved across cortical regions, yet differences in neuromodulatory receptor expression, the density of specific cell types, or in the specifics of local connectivity can alter the influence of neuromodulatory input on the pattern of neural population activity. Indeed, the density of SOM neurons is increased relative to PV neurons in association cortex (Kim et al., 2017; Dienel et al., 2021), suggesting that the population-level computations that SOM neurons participate in, and thus the relationship between arousal state and local network state, may differ across the cortical processing hierarchy.

Here, we hypothesized that arousal-related modulation of SOM neurons, and of local neural activity, would be specialized across cortical regions to match different arousal-related demands on local computation. We examined the effects of arousal state on activity in SOM and Non-SOM neurons in the primary auditory cortex (AC), and in the posterior parietal cortex (PPC). AC is a primary sensory region of the cortex, where the relationship between arousal and neural activity has been well studied (Schneider et al., 2014; Zhou et al., 2014; McGinley et al., 2015; Bigelow et al., 2019; Yavorska and Wehr, 2021). PPC is an association-level region that participates in flexible sensorimotor transformations (Fitzgerald et al., 2011; Harvey et al., 2012; Morcos and Harvey, 2016; Licata et al., 2017; Tseng et al., 2022). In PPC, task engagement is known to impact the structure of local population activity (Runyan et al., 2017; Pho et al., 2018; Valente et al., 2021), though relatively little is known about the specific contribution of generalized increases in arousal to the activity of PPC. Outside of a task context, firing rates of neurons in PPC are positively correlated with arousal level (Stitt et al., 2018), but the effects of arousal on specific inhibitory neuron types within PPC are not known. In the current study, we have revealed different relationships among arousal state, the structure of local population activity, and information coding in AC and PPC, suggesting that the effects of arousal on local processing are specialized across the cortical hierarchy.

## Materials and Methods

### Experimental design and statistical analysis

All pairwise comparisons were done with two-sided paired or unpaired permutation (i.e., randomization) tests with 10,000 iterations as indicated, where *p* < 0.0001 indicates the highest significance achievable given the number of iterations performed. Given that the exact *p* value is unknown in these cases, *p* values of the highest significance are reported as such rather than as an exact value. All permutation tests were performed for differences in means. For statistical comparisons involving more than two groups, we used Kruskal–Wallis (nonparametric ANOVA) and used unpaired permutation tests *post hoc* to determine which groups differed from each other. Data fell into natural groupings by (1) brain area (AC or PPC) and by (2) cell-type (SOM or Non-SOM), as indicated by expression of the red fluorophore, tdTomato. All bar plots show the mean and bootstrapped 95% confidence intervals using 1000 iterations unless otherwise indicated. When multiple comparisons were made between groups, significance thresholds were Bonferroni corrected. Sample sizes were chosen based on previous studies comparing population activity dynamics across brain areas or cell types (Runyan et al., 2017; Khan et al., 2018).

### Animals

All procedures were approved by the University of Pittsburgh Institutional Animal Care and Use Committee. Homozygous SOM-Cre mice (Sst-IRES-Cre; stock #013044) were crossed with homozygous Ai14 mice (RCL-tdT-D; stock #007914) obtained from The Jackson Laboratory, and all experiments were performed in the F1 generation, which expressed tdTomato in SOM^+^ neurons. Mice were group housed in cages with between two and four mice. Adult (8–24 weeks) male and female mice were used for experiments (four male, two female). Mice were housed on a reversed 12 h light/dark cycle, and all experiments were performed in the dark (active) phase.

Mice were anesthetized with isoflurane (4% for induction, and 1–2% maintenance during surgery), and mounted on a stereotaxic frame (David Kopf Instruments). Ophthalmic ointment was applied to cover the eyes (Henry Schein Medical). Dexamethasone was injected 12–24 h before surgery, and carprofen and dexamethasone (Covetrus) were injected subcutaneously immediately before surgery for pain management and to reduce the inflammatory response. Two circular craniotomies, each of 2 mm diameter, were made over left AC and PPC (centered at 2 mm posterior and 1.75 mm lateral to bregma). For AC, the craniotomy was centered on the temporal ridge, and the posterior edge was aligned with the lambdoid suture. Two millimeter biopsy punches were used to outline the circumference of the window before drilling.

One to four evenly spaced ∼60 nl injections of the AAV1-synapsin-GCaMP6f (stock #100837, Addgene) that had been diluted to a titer of ∼1 × 10^12^ viral genomes/ml using sterile PBS were made in each cranial window, centered in each craniotomy. A micromanipulator (QUAD) was used to target injections ∼250 μm under the dura at each site, where ∼60 nl of virus was pressure injected over 5–10 min. Pipettes were not removed until 5 min postinjection to prevent backflow. Dental cement (Parkell) sealed a glass coverslip (3 mm) over a drop of Kwik Sil (World Precision Instruments) over the craniotomy. Using dental cement, a one-sided titanium headplate was attached to the right hemisphere of the skull. After mice had recovered from the anesthesia, they were returned to their home cages, and received oral carprofen tablets (Bio-Serv) for 3 d postsurgery.

### Experimental setup

#### Two-photon microscope

Images were acquired using a resonant scanning two-photon microscope (Ultima Investigator) at a 30 Hz frame rate and 512 × 512 pixel resolution through a 16× water-immersion lens (16×/0.8 numerical aperture; model CF175, Nikon). On separate days, either AC or PPC was imaged at a depth between 150 and 300 μm, corresponding to layers 2/3 of cortex. For AC imaging, the objective was rotated 35–45° from vertical, and for PPC imaging, it was rotated to 5–15° from vertical, matching the angle of the cranial window implant. Fields of view were 500 μm^2^ and contained 187 ± 95 neurons, 20 ± 10 (mean ± SD) of which were classified as SOM neurons. Excitation light was provided by a femtosecond infrared (IR) laser (Insight X3, Spectra-Physics) tuned to 920 nm. Green and red wavelengths were separated through a 565 nm low-pass filter before passing through bandpass filters (catalog #ET525/70 and #ET595/50, Chroma). PrairieView software (version 5.5; Bruker) was used to control the microscope.

#### Behavioral monitoring

Running velocity was monitored on pitch and roll axes using two optical sensors (model ADNS-98 000, Tindie) held adjacent to the spherical treadmill. A microcontroller (Teensy 3.1, Adafruit) communicated with the sensors, demixing their inputs to produce one output channel per rotational axis using custom code. Outputs controlling the galvanometers were synchronized with running velocity using a digital oscilloscope [WaveSurfer, Janelia Research Campus, Howard Hughes Medical Institute (HHMI)].

Pupil images were acquired at 1280 × 1024 pixels, at 10 Hz from an IR camera focused on one eye [Flea3 FL3-U3-13Y3M-C one-half inch Monochrome USB 3.0 Camera, with 1.0× SilverTL Telecentric Lens (field of view, 6.74 × 5.39 mm), Edmund Optics]. The pupil was illuminated by the IR light emitted by the two-photon laser and required no additional IR illumination. Movies were acquired with the MATLAB Image Acquisition Toolbox (MathWorks). Pupil area was determined in each pupil movie frame *post hoc* using custom MATLAB code (MathWorks). The pupil was constricted by controlling ambient illumination with an array of LCD screens (LP097QX1, LG Display) to maintain a moderate pupil area baseline from which increases and decreases in area could be measured.

#### Experimental protocol

Imaging began 3–5 weeks postsurgery once robust expression of the GCaMP6f virus was observed. In each imaging session, GCaMP6f fluorescence changes were imaged in SOM (tdTomato^+^) and Non-SOM neurons, while mice ran freely on a spherical treadmill. In the spontaneous context, no sensory stimuli were delivered, while in the passive-listening context, location-varying sound stimuli were presented (see Materials and Methods, subsection Sound stimuli). Spontaneous and passive listening contexts lasted ∼25–50 min each. Imaging alternated between AC and PPC across days. Multiple imaging sessions were performed in each cranial window, focusing at slightly different depths and lateral/posterior locations within the imaging windows across sessions. AC and PPC were each imaged in six mice (biological replicates). Each cranial window was imaged up to 11 times (technical replicates). Imaging from a given cranial window was suspended when we observed nuclear inclusion in two or more cells in the field of view, which indicates an overexpression of GCaMP6f.

#### Sound stimuli

Four magnetic speakers were positioned in a semicircular array (model MF1-S, Tucker-Davis), centered on the mouse’s head. The speakers were positioned at −90°, −30°, +30°, and +90° from the midline in azimuth and driven by MATLAB through a digital/analog converter (National Instruments). Speakers were calibrated to deliver similar sound levels (∼70 dB) in a sound isolation chamber using a random incidence microphone (model 4939, Brüel & Kjær). During passive listening, 1 or 2 s dynamic ripples (broadband stimuli created in MATLAB by summing 32 tones spaced across 2–32 kHz, which fluctuated at 10–20 Hz; Elhilali et al., 2004) were presented from one of eight locations. Four of the sound locations corresponded to the locations of the four speakers (−90°, −30°, +30°, +90°), while the other four sound locations (−60°, −15°, +15°, +60°) were simulated using vector-based intensity panning, where the same sound stimulus was delivered to two neighboring speakers simultaneously, scaled by a gain factor (Runyan et al., 2017). Dynamic ripples were chosen to optimally drive populations of neurons in auditory cortex with diverse frequency tuning preferences. Each sound repeated three times at one location before switching to another. Each ripple played from each of the eight locations in randomized order, with a 240 ms gap between each sound. Output controlling the audio speakers was recorded along with two-photon imaging galvo and running velocity using WaveSurfer (Janelia Research Campus, HHMI), and these signals were aligned offline.

### Data processing

Imaging datasets from 24 AC fields of view and 20 PPC fields of view were included from six mice. We excluded any datasets with significant photobleaching or more than two filled cells. We also excluded any AC or PPC dataset from analysis if fewer than one-third of neurons that were significantly responsive (according to our definition in subsection Sound responsiveness) to at least one sound location, as we were interested in the effect of arousal on both spontaneous and sound-evoked responses. For AC datasets, we analyzed single-cell responses to pure tones on a subset of fields of view from each mouse, and then anatomically aligned all fields of view from datasets collected from each window, to ensure each field of view lay in a region representing tone frequencies in the sonic range of the tonotopic axis of primary auditory cortex. We eliminated any datasets where >50% of tone-responsive neurons had a preferred frequency that was in the ultrasonic range (>20 kHz), as well as any fields of view that were aligned anterior to a field of view where this was observed, to assure that we were seeing sound responses in the range of frequencies primarily represented by our dynamic ripples (described in subsection Sound stimuli). We collected wide-field fluorescence responses to pure tones in all AC cranial windows and observed pure-tone responses in the sonic range for all AC windows; however, the extent of the viral expression within windows was too spatially limited to allow for mapping of specific regions.

#### Image processing

For each field of view, the raw calcium movies collected during the spontaneous activity and passive listening contexts were concatenated before motion correction, cell body identification, and fluorescence and neuropil extraction. These processing steps were performed using Suite2p 0.9.3 in Python (Pachitariu et al., 2017). Suite2p first registered images to eliminate brain motion, and clustered neighboring pixels with similar time courses into regions of interest (ROIs). ROIs were manually curated using the Suite2p graphical user interface (GUI), to ensure that only cell bodies, as opposed to dendritic processes, were included in analysis, based on morphology. Cells expressing tdTomato (SOM cells) were identified using a threshold applied in the Suite2p GUI based on mean fluorescence in the red channel after bleed-through correction applied by the Suite2p cell detection algorithm, along with manual correction. For each ROI, Suite2p returned a raw fluorescence time series, as well as an estimate of neuropil fluorescence that could contaminate the signal. For each cell, we scaled the neuropil fluorescence by a factor by 0.7 and subtracted this time series from the raw fluorescence time series of the ROI to obtain a neuropil-corrected fluorescence signal for each selected cell.

#### Δ*F*/*F* and deconvolution

**Figure 1.**
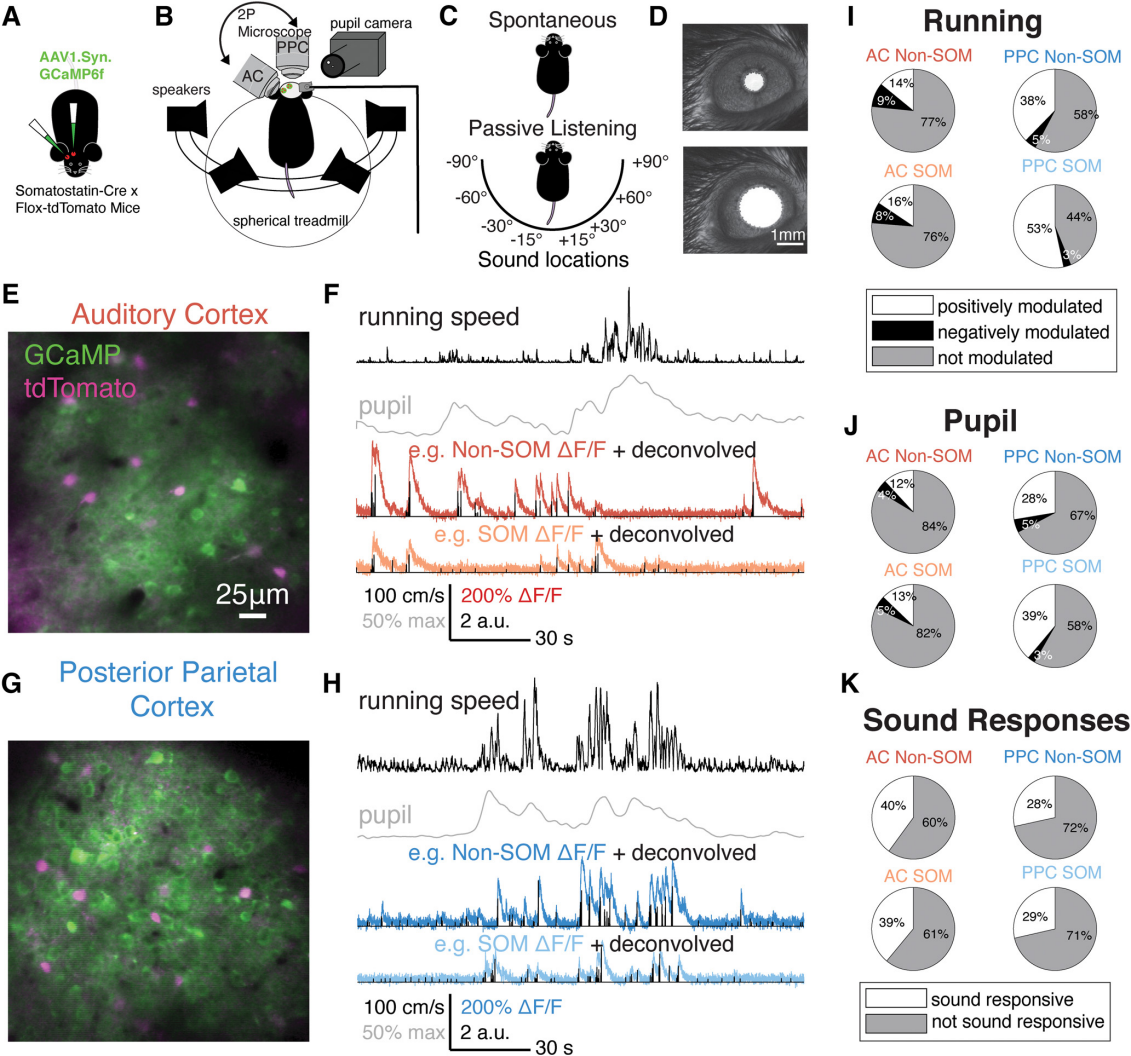
Imaging spike-related activity in SOM and Non-SOM neurons during behavioral state transitions. ***A***, Viral injections and cranial windows were made over AC and PPC in each SOM-tdTomato mouse. ***B***, In imaging sessions, mice were headfixed over a spherical treadmill and allowed to run voluntarily. Four speakers arranged around the head presented sound stimuli. An infrared camera was used to image the pupil, and a rotating two-photon microscope was focused on either AC or PPC on a given imaging day. ***C***, Each imaging session included spontaneous and passive listening contexts, without or with randomly presented sound stimuli from each of eight locations, respectively. ***D***, Pupil area was monitored via the pupil camera; the scale bar in the bottom image applies to top (constricted pupil) and bottom (dilated pupil). ***E***, Example field of view from auditory cortex, with intermingled tdTomato^+^/SOM^+^ (magenta) and tdTomato^–^/SOM^–^ neurons, coexpressing GCaMP6f (green). ***F***, Example aligned behavioral and neural signals collected during the imaging session in ***E***, including running speed (in cm/s), normalized pupil area, dF/F from a Non-SOM (red) and SOM neuron (orange), each overlaid with the deconvolved estimated spike rates of the neuron. ***G***, As in ***E***, for an example posterior parietal cortex field of view. ***H***, As in ***F***, for the PPC field of view in ***G***. ***I***, Proportions of Non-SOM and SOM neurons in AC (left) and PPC (right) with significant positive (white), negative (black), or no modulation (gray) by the of the mouse running. AC Non-SOM, *N* = 2645; AC SOM, *N* = 359; PPC Non-SOM, *N* = 4719; PPC SOM, *N* = 525. ***J***, As in ***I***, for pupil dilation. ***K***, Proportion of Non-SOM and SOM neurons in AC and PPC that were significantly sound responsive to at least one location (white) or not significantly sound responsive (gray).

Once the neuropil corrected fluorescence was obtained for each neuron, we calculated ΔF/F for each cell in each frame by calculating (*F* – *F*_baseline_)/*F*_baseline_ for each frame, where *F* is the fluorescence of a given cell at that frame and *F*_baseline_ was the eighth percentile of the fluorescence of that cell spanning 450 frames before and after (∼15 s each way, 30 s total). Δ*F*/*F* time series were then deconvolved to estimate the relative spike rate in each imaging frame using the OASIS toolbox (Friedrich et al., 2017). We used the AR1 FOOPSI algorithm and allowed the toolbox to optimize the convolution kernel, baseline fluorescence, and noise distribution. A threshold of 0.05 a.u. was applied to remove all events with low magnitude from deconvolved activity time series. All analyses were performed with both Δ*F*/*F* and deconvolved activity, and showed the same trends. Outside of Figure 1*F* and 1*H*, only results using deconvolved activity are shown.

### Single-cell modulation by sound stimuli, running behavior, and pupil size

#### Sound responsiveness

The deconvolved activity of each neuron was *z* scored across its entire time series and trial averaged. For each sound location, we then calculated the sound-evoked response as the difference between the mean activity during the sound presentation and the mean activity in the 240 ms before sound onset. We then compared the evoked sound responses to shuffled distributions, where the activity of each cell was shifted randomly by at least 5 s in time relative to sound location time series, and the sound-evoked response was recalculated. This was repeated 1000 times. A neuron was considered to be sound responsive if it had a sound-evoked response in at least one sound location that was greater than the 97.5 percentile of the shuffled distribution.

#### Running bouts and modulation

Running bout onsets were defined as transitions in speed from <10 to >10 cm/s, and required that the mean running speed in the 1 s following the transition was three times greater than the 1 s before running bout onset, and that the mouse maintained a minimum speed of 15 cm/s for the following 2 s.

Running modulation was calculated as the difference in mean activity of a cell in the 1 s before running bout onset and the mean activity of a cell in the 3 s window following running bout onset. A shuffling procedure was applied to determine which cells were positively, negatively, and not modulated by running. The activity of each cell was shifted randomly by at least 5 s in time relative to running speed time series, and for 1000 time-shifted iterations, running modulation was recalculated. Positively modulated neurons had positive running modulation values higher than the 97.5 percentile of the shuffled distribution of that cell. Negatively modulated cells had negative running modulation values lower than the 2.5 percentile of the shuffled distribution of that cell. All other cells were considered to not be modulated by running speed increases.

#### Pupil dilation events and modulation

Pupil area was normalized to its maximum across the imaging session. To identify pupil dilation events, we first identified all local maxima of the pupil area. We then found the point before this where the derivative of pupil area was zero. We included events where the time from the inflection point to the local maximum was at least a 40% increase in pupil area and that the change from the inflection point to the local maximum was <1 s, and that the local maximum was at least 50% of the maximum total area by the pupil during that imaging session. We considered each inflection point to be the onset of dilation events.

To capture all pupil dilation-related activity, which had a slower time course than running (see Fig. 4), we calculated pupil modulation for each neuron as the difference between the mean activity in the 1 s time window before dilation event onset and the mean activity in the 5 s time window after dilation event onset. We applied the same shuffling procedure as described for running modulation (subsection Running bouts and modulation) to determine which neurons were positively, negatively, and not modulated by pupil dilation events.

### Arousal states

#### Defining low and high arousal states based on pupil area

K-means clustering was applied to the full pupil area time series, which included both spontaneous activity and passive listening contexts, to classify each pupil area measurement as low, transitional, or high arousal. Each pupil area time series was the mean normalized using the following equation:

$$\frac{X-\bar{X}}{\bar{X.}}$$

The Manhattan (called City Blocks) distance metric was applied to define two centroid clusters that served as the high arousal and low arousal groups. Transition periods included timepoints when the absolute difference in distance to the high arousal and low arousal centroids was <0.05.

#### Arousal modulation index

The arousal modulation index (AMI) was calculated for each neuron using the following equation:

$$\frac{{FR}_{hi}{\text{−}FR}_{lo}}{{FR}_{hi}{\text{+}FR}_{lo}},$$

where FR_hi_ is the mean response of the neuron in the high arousal state and FR_lo_ is the mean response of the neuron in the low arousal state. We first maximum normalized the deconvolved activity trace of each neuron across the entire time series. To calculate FR_hi_ (or FR_lo_), we summed the activity from the high (or low) arousal state and divided by the total time spent in the high (or low) arousal state in the spontaneous context. This index could vary continuously between −1 and +1, where negative values indicate higher activity in the low arousal state, and positive numbers indicate higher activity during the high arousal state.

### Encoding models

We used an encoding model to disentangle the contributions of pupil size and running speed to the activity of neurons in AC and PPC. In the generalized linear model (GLM), the time-dependent effects of all measured external variables on the activity of each neuron were estimated (Pillow et al., 2008; Runyan et al., 2017). The following three classes of predictors were used in different combinations to quantify their contributions to neuronal activity: running, pupil size, and sound stimulus predictors. We used a Bernoulli-based GLM to weight various combinations of predictors based on these variables to predict the binarized activity of each neuron (time series of relative spike rates were thresholded at 0.05). The encoding model is fully described in our previous work (Runyan et al., 2017).

#### Pupil size and running predictors

Running velocity was measured at a higher time resolution than imaging and was binned to match the sampling rate of two-photon images (30 Hz). We included the velocity along the pitch and roll axes of the treadmill (relative to the mouse body axis). Running velocity measurements were separated into the following four channels: (1) forward, (2) reverse, (3) left, and (4) right directions based on rotation along these axes. Running velocity changes could both precede or follow the activity of individual neurons, so time series of running velocity were convolved with four evenly spaced Gaussian basis functions (240 ms half-width at half-height) extending 1 s both forward and backward in time (eight basis functions total for each running direction: forward, reverse, left, and right). Changes in pupil area were modeled similarly. Because pupil area changes on a slower timescale, the pupil area trace was convolved with 16 evenly spaced Gaussian basis functions 4 s forward and backward in time to allow for either prediction or response to pupil area changes.

#### Sound stimulus predictors

Sound stimuli were delivered from specific sound locations in the passive listening context. For sound stimulus onsets at each of the possible sound locations, 12 evenly spaced Gaussian basis functions (170 ms half-width at half-height) extended 2 s forward in time from each sound onset. First, second, and third repeats were represented separately because of potential adaptation-related effects. This resulted in 12 basis functions per repeat per sound location × three repeats × eight locations for 288 sound predictors.

#### GLM fitting and cross-validation procedures

All predictors were maximum normalized before the fitting procedure. The β-coefficients for the predictors were fitted to the activity of each neuron individually, using the glmnet package in R (Friedman et al., 2010) with elastic-net regularization, which smoothly interpolated between L_1_ and L_2_ type regularization according to the value of an interpolation parameter α, such that α = 0 corresponded to L_2_ and α = 1 corresponded to L_1_. We selected α = 0.25.

Trials were randomly split into training (70% of trials) and testing (remaining 30% of trials) sets, while balancing the distribution of sound locations. Fitting was performed on the training set, and within each training dataset, cross-validation folds (3×) were also preselected so that sound locations were evenly represented. Model performance (see below, subsection GLM model performance) was assessed on the test set. Each model was thus fitted and tested on entirely separate data to prevent overfitting from affecting results. This train/test procedure was repeated 10 times, with random subsamples of the data included in train and test segments. The overall performance of each model was assessed as its mean across all 10 iterations.

#### GLM model performance

Each model’s performance for each cell was defined as the fraction of explained deviance of each model (compared with the null model). In the null model, only a constant (single parameter) was used to fit the neuron’s activity and no time-varying predictors were included. First, we calculated the deviance of the null and behavior model variants (see Materials and Methods, subsection Running and pupil contribution). For each model, the fraction of null model deviance explained by the model (*d*) was then calculated [(null deviance – model deviance)/null deviance]. Deviance calculations were performed on a test dataset (30% of the data), which had not been included in the fitting procedure, and this train/test procedure was repeated 10 times on randomly subsampled segments of the data.

#### Running and pupil contribution

To identify the unique and separable contributions of running and pupil area to activity of SOM and Non-SOM neurons, we fit the following three separate models: (1) full behavior model, (2) “no-pupil” model, and (3) “no-running” model. In the full behavior model, all running, pupil, and sound predictors were included to predict the activity of each neuron. The no-pupil model did not include the pupil predictors, and the no-running model did not include the running predictors. Importantly, this analysis captures only the unique ways that pupil size and running can explain neural activity, where one cannot compensate for the contribution of the other.

We estimated the contribution of pupil or running to the activity of a neuron that could not be compensated for by the other variables, by comparing the model performance (fraction deviance explained, see Materials and Methods, subsection GLM model performance) in the full behavior versus no-pupil or no-running models. The “running contribution” was calculated as the difference in fraction deviance explained of the full model and fraction deviance explained of the no-running model *d_fb_* – *d_nr_*, where *d_fb_* is the full behavior deviance and *d_nr_* is the no-running deviance. The “pupil contribution” was calculated as the difference in fraction deviance explained of the full model and fraction deviance explained of the no-pupil model *d_fb_* – *d_np_*, where *d_np_* is the no-pupil deviance.

### Decoding

We used a population decoder to compare the sound location information contained in AC and PPC population activity, and its modulation with arousal state. The details of the decoder that we built to estimate the information about sound stimulus location have been previously described (Runyan et al., 2017). Briefly, for each trial we decoded sound stimulus location from single-trial population activity by computing the probability of external variables (sound location left/right category) given population activity. We used Bayes’ theorem, relying on population response probabilities estimated through the full behavior GLM and its predictors in that trial, to compute the posterior probability of each possible sound location stimulus. The decoder was “cumulative” in time, as for each time point *t*, it was based on all imaging frames from the initiation of the trial through time *t*. The decoded stimulus location at each time *t* was defined as the stimulus location with the maximum posterior probability, based on individual neurons or on a population of simultaneously imaged neurons. The population could include SOM, Non-SOM, or the “best” neurons. Non-SOM neurons were randomly subsampled 10 times, matching the sample size of SOM neurons in each iteration. The best neurons were selected as the *n* individual neurons with the best decoding performance, where *n* is the number of SOM neurons simultaneously imaged. Decoder performance was calculated as the fraction of correctly classified trials at each time point.

To compare decoder performance in low and high arousal states, trials were classified as “low” or “high” arousal based on normalized pupil area. Only the first sound repetition of each trial was used. Trials were randomly subsampled in the test set to ensure an even distribution of low and high arousal trials, and sound locations. This random subsample was repeated 10 times.

### Sound location sensitivity

To assess the location sensitivity of sound-related activity in SOM and Non-SOM neurons in AC and PPC, trial-averaged responses were used to calculate the “location sensitivity index” (LSI), based on vector averaging in the preferred sound direction, as follows:

$$LSI=\sqrt{\left(\overset{n}{\underset{i=1}{\sum }}R\left({\text{Θ}}_{i}\right)cos{\left(2{\text{Θ}}_{i}\right)}^{2}+\overset{n}{\underset{i=1}{\sum }}R\left({\text{Θ}}_{i}\right)sin{\left(2{\text{Θ}}_{i}\right)}^{2}\right)}/\overset{n}{\underset{i=1}{\sum }}R_{i},$$

where *R* is the average response during the sound location presentation, and Θ is the sound location from −90° to +90°, indexed by *i* = 1 to *n* (eight possible locations). LSI can vary continuously from 0 (unselective) to 1 (selectively responding to only one sound location). Datasets that did not have a minimum of 20 trials for each of the eight sound locations were excluded. Importantly, sounds were played from free-field speakers located at different angles relative to the interaural axis. A high LSI could correspond to true sound location selectivity based on interaural level differences, or to the specific intensity tuning of a neuron, because of different sound intensities impinging on the two ears.

### Population-level analyses

#### Defining population activity axes related to sound location and arousal

To determine to what degree sound stimuli and arousal were driving population activity independently of each other in AC and PPC, we computed two axes for each imaging session: a sound location axis and a pupil axis. The sound location axis was defined as the axis in population activity space that connects the mean response on −90° and +90° trials during the passive listening behavioral context, while the pupil axis was defined as the axis that connects the mean population activity during “low arousal” and “high arousal” periods during the spontaneous behavioral context, as defined by our pupil-clustering algorithm. These definitions are analogous to how the “attention axis” is computed in primates (Cohen and Maunsell, 2010; Mayo et al., 2015; Cowley et al., 2020). We then measured the angle between the two vectors with a value from 0° to 90°, where 0° indicates linear dependence between the two subspaces and 90° indicates orthogonality.

#### Computing signal and noise correlations

We calculated noise correlations as fluctuations around mean sound responses; therefore, we only included neurons that had significant sound responses (Fig. 1*K*; see also Materials and Methods, subsection Sound Responsiveness). Because it was rare for the pupil of a mouse to remain either in the high or low arousal cluster for the entire duration of a trial including all three sound repeats, we focused our analysis instead on the first repeat of a trial. We binned the *z*-scored, deconvolved activity of each neuron into 15-frame (∼500 ms) bins following sound onset. For each trial classified as high or low arousal, the mean sound response of each cell to all matching sound location presentations (including low, high, and unclassified trials) was subtracted and trials were concatenated. We computed partial Pearson correlations, discounting the effect of running speed (MATLAB function partialcorr), on these traces. Because the ratio of low to high trials was variable across imaging sessions, we subsampled 10 times to balance for matching numbers of high and low trials at each sound location. We only considered imaging sessions in which there were at least 50 matched trials in low and high arousal clusters.

### Histology

After all imaging sessions had been acquired, each mouse was transcardially perfused with saline and then 4% paraformaldehyde. The brain was extracted, cryoprotected, embedded, frozen, and sliced. Once slide mounted, we stained brains with DAPI to be able to identify structure. We used anatomic structure to verify the locations of our injections in AC and PPC.

## Results

To determine whether the effects of arousal on local activity are conserved across sensory and association cortices, we compared spontaneous and sensory-evoked activity in AC and PPC in six mice of both sexes, during spontaneous shifts in the arousal state of the animals. We used two-photon calcium imaging in superficial cortex to measure the spike-related activity of neurons positive and negative for the red fluorophore tdTomato, which was expressed transgenically in SOM-positive neurons (Madisen et al., 2010; Taniguchi et al., 2011). We virally expressed the genetically encoded calcium indicator GCaMP6f in all layer 2/3 neurons of AC and PPC in each mouse (Chen et al., 2013).

### Neural activity was modulated by sound stimuli and by behavioral correlates of arousal

During each imaging session, we focused the microscope on either AC or PPC and imaged neural activity in two contexts. During the “spontaneous” context, the mouse ran freely on the spherical treadmill in the absence of sensory stimulation. During passive listening, sounds were presented from each of eight possible locations (Fig. 1*B*,*C*). In both contexts, mice were head fixed and allowed to run voluntarily on a spherical treadmill. To track the behavioral state, running velocity and pupil area of the mouse were recorded throughout imaging sessions (Fig. 1*A–H*).

We first examined how changes in pupil area and running speed corresponded with changes in the activity of individual neurons during the spontaneous context. Throughout imaging sessions, mice transitioned between behavioral states that reflect different arousal states: stillness and running, and pupil constriction and dilation (Fig. 1*F*,*H*). To quantify the effect of behavioral state transitions on the activity of individual neurons, we identified timepoints during the spontaneous context when either running speed or pupil area increased (Materials and Methods, subsections Running bouts and modulation and Pupil dilation events and modulation). The frequency of running bouts and dilation events was similar during PPC and AC imaging sessions (*p* = 0.59 and *p* = 0.65, respectively, permutation test, here and throughout unless otherwise noted; see Materials and Methods, subsection Experimental design and statistical analysis). Mice initiated running bouts at a mean rate of 0.915 bouts/min (bootstrapped 95% confidence interval of the mean, 0.749–1.11, here and throughout, unless otherwise indicated), and pupil dilations at 0.981 dilations/min (bootstrapped 95% confidence interval, 0.857–1.12). We observed no difference between AC (*N* = 24) and PPC (*N* = 20) imaging sessions when considering pupil area and running speed during passive listening or spontaneous contexts (passive running speed, *p* = 0.20; spontaneous running speed, *p* = 0.52; passive pupil area, *p* = 0.43; spontaneous pupil area, *p* = 0.73). However, pupil area and running speed tended to be higher in general during the passive listening context (pupil, *p* = 0.0020; running, *p* < 0.0001; paired permutation test, *N* = 44 datasets; Extended Data Fig. 1-1).

10.1523/ENEURO.0136-23.2023.f1-1 Figure 1-1 Characterizing running behavior and pupil size across contexts. ***A***, Left, Mean running speed in the spontaneous and passive listening behavioral contexts. Right, Mean running speed during the passive listening context plotted against the mean running speed during the spontaneous context, for each imaging session, *p* = 0.0003. ***B***, Left, Mean, maximum normalized pupil area during the spontaneous and passive listening contexts. Right, Mean pupil area in the two contexts for each imaging session, *p* = 0.002. ***C***, Left, Mean time spent running <15 cm/s in spontaneous and passive listening contexts. Right, Time spent running <15 cm/s during the two contexts, for each imaging session, *p* < 0.0001. ***D***, Left, Mean time spent running >15 cm/s in spontaneous and passive listening contexts. Right, Time spent running >15 cm/s in the two contexts, for each imaging session. *N* = 44 imaging sessions for all panels. Error bars indicate bootstrapped 95% confidence intervals. ***p < 0.001; **p < 0.01. Download Figure 1-1, TIF file.

To determine how behavioral state affected neuronal activity, we next examined the activity of individual neurons during transitions from stationary to running, in the “spontaneous context,” when no sound stimuli were presented. We compared mean activity of each neuron (deconvolved estimated spike rates, here and throughout) in the 3 s time window following running bout onset to the 1 s time window before running bout onset (Fig. 1*I*; see Materials and Methods, subsection Running bouts and modulation). Pupil size modulation was calculated similarly, based on transitions in pupil area from constricted to dilated (Fig. 1*D*,*J*; Materials and Methods, subsection Pupil dilation events and modulation). We compared the running and pupil modulation of each neuron to shuffled distributions, where activity and behavioral data were time shifted by random intervals, and classified each neuron as positively, negatively, or not modulated by behavioral state transition. Larger proportions of the SOM and Non-SOM populations were positively modulated by both pupil dilations and running bout onsets in PPC than in AC (Fig. 1*I*,*J*), suggesting that the effects of arousal on spontaneous activity are not uniform across areas. Among the groups considered, the PPC SOM population had the greatest proportion of neurons modulated by running speed and pupil dilation (Fig. 1*I*,*J*).

In the passive listening context, sound stimuli were presented from each of eight locations, centered on the head of the mouse (Fig. 1*B*,*C*; see Materials and Methods, subsection Sound stimuli). We chose to manipulate sound location because of the role of PPC in spatial auditory processing during active behaviors (Nakamura, 1999). To determine whether each neuron was generally sound responsive, we computed the mean difference in activity during sound presentations and the prestimulus periods and compared with a shuffled distribution (Materials and Methods, subsection Sound responsiveness). We defined neurons as sound responsive if they responded to at least one sound location, more than would be expected from a random distribution obtained by shuffling. As expected, a greater proportion of AC SOM and Non-SOM neurons was sound responsive compared with PPC (39% of AC SOM and 40% of AC Non-SOM; 29% of PPC SOM and 28% of PPC Non-SOM neurons; Fig. 1*K*). The fraction of sound responsive neurons in AC is similar to the sparse sound encoding population described by others in layer 2/3 of AC (Hromádka et al., 2008). Furthermore, sound-evoked responses in AC Non-SOM neurons were lower when mice were running than when they were stationary (*p* < 0.05, Extended Data Fig. 1-2), as has been reported on extensively by others (Schneider et al., 2014; Bigelow et al., 2019; Yavorska and Wehr, 2021). In PPC, sound-evoked responses were not affected by running behavior.

**Figure 2.**
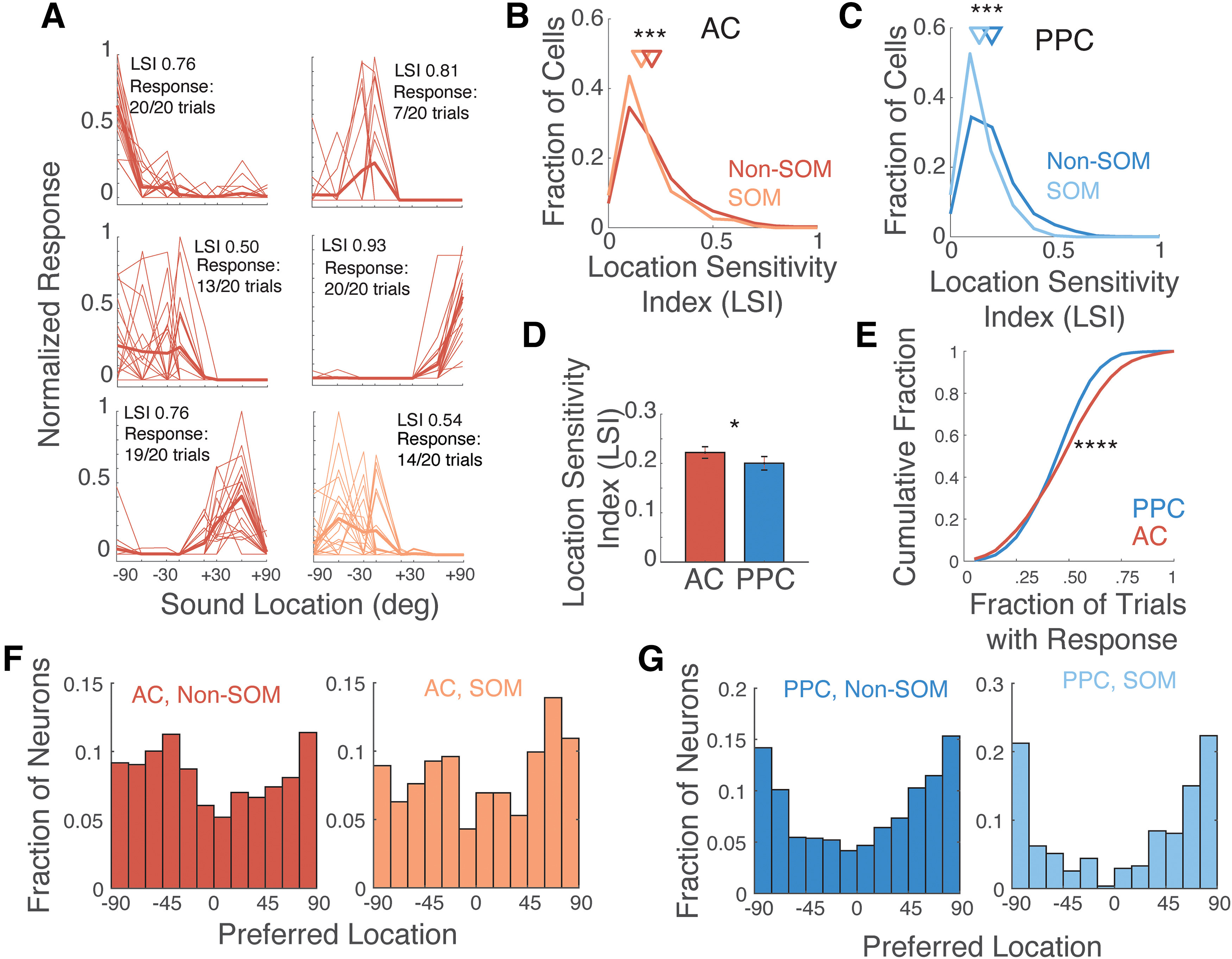
Sound location sensitivity in AC and PPC. ***A***, Six example neurons with diverse sound location preferences, ranging from ipsilateral (−90° to 0°) to contralateral (0° to +90°) locations. Thin lines, Mean response during sound presentation of individual trials; thick lines, trial average. The LSI and response reliability are reported for each example cell. Red, Example Non-SOM neurons; orange, example SOM neuron. ***B***, Distribution of LSI for Non-SOM (mean, 0.20; 95% CI, 0.20, 0.21; *N* = 2455) and SOM (mean, 0.19; 95% CI, 0.18, 0.19; *N* = 354) neurons in AC. Triangles indicate population means. ***C***, As in ***B***, for PPC Non-SOM (mean, 0.20; 95% CI, 0.19, 0.20; *N* = 2553) and SOM (mean, 0.13; 95% CI, 0.12, 0.14; *N* = 298). ***D***, Mean location sensitivity index in AC and PPC. ***E***, Cumulative distributions of response reliability, the fraction of sound-responsive trials, in AC and PPC. ***F***, Histograms of the preferred locations of all AC Non-SOM and SOM neurons with LSI values >0.05. ***G***, Histograms of the preferred locations of all PPC Non-SOM and SOM neurons with LSI values >0.05. *p < 0.05, ***p < 0.001, ****p < 0.0001. Error bars indicate the SEM.

10.1523/ENEURO.0136-23.2023.f1-2 Figure 1-2 Sound-evoked responses during periods of stillness and running. ***A***, Left, Sound-evoked activity of sound-responsive AC Non-SOM neurons (Fig. 1*K*; Materials and Methods), in response to the preferred sound location. Gray, Responses during trials when mice were running <15 cm/s; black, responses during trials when mice were running >20 cm/s. Sound-evoked activity was baseline subtracted using the preceding 240 ms window for ease of display. Black arrow indicates sound onset. Right, Mean sound-evoked activity during the first 500 ms of sound presentation during still and running periods, *n* = 432, *p* = 0.017 (paired permutation test with 10,000 iterations here, and throughout). Subsampling to match locomotion condition and repetition was repeated 10 times for each sound location. ***B***, As in ***A***, for AC SOM neurons, *n* = 59. ***C***, As in ***A*** and ***B*** for PPC Non-SOM neurons, *n* = 901. ***D***, As in ***A–C*** for PPC SOM neurons, *n* = 112. *p < 0.05. Download Figure 1-2, TIF file.

### Sound location coding in AC and PPC

To characterize the sound location sensitivity of responses in AC and PPC during the passive listening context, we computed the sound Location Sensitivity Index (LSI) of each neuron (Fig. 2*A–D*; Materials and Methods, subsection Sound location sensitivity). It is important to note that in the free-field sound stimulation configuration, we cannot distinguish true sound location selectivity from differences in sound intensity tuning. In both AC and PPC, Non-SOM neurons were more sensitive to sound location than SOM neurons (*p* < 0.0001; Fig. 2*B*,*C*). PPC neurons overall were less sensitive than AC neurons (Fig. 2*D*), and PPC sound responses were less reliable than AC sound responses (*p* < 0.001; Fig. 2*E*). Interestingly, among neurons with sound location preferences, PPC sound responses were biased toward the lateral locations at +90° (contralateral) and −90° (ipsilateral), while the distribution of sound location preferences in AC neurons was more uniform (Fig. 2*F*,*G*). Based on these differences in sound location sensitivity and response reliability, we expected population activity to encode sound location more accurately in AC than PPC.

**Figure 3.**
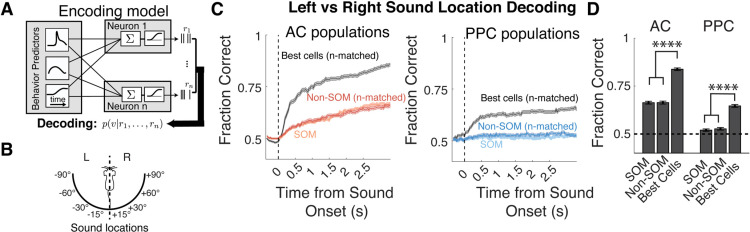
Decoding sound location from AC and PPC population activity. ***A***, The encoding model was trained on all trials that included all arousal levels, and inverted using Bayes’ rule to compute the posterior probability of auditory stimuli given the activity of the neural population in AC and PPC. ***B***, Schematic of the discrimination being performed by the decoder, classifying sound stimuli as occurring from the left or right of the mouse. ***C***, Left, Mean cumulative population decoder performance (across datasets) when classifying left versus right locations, when based on best cells, defined as individual Non-SOM cells or SOM cells with the highest decoding performance. Best cells and Non-SOM cells in each dataset were subsampled to match the *N* of the SOM neuron population within each imaging field of view. Right, Population decoder performance using PPC neurons. Chance performance is 0.5. ***D***, Mean decoder performance using all timepoints in the trial (equivalent to the final timepoints in ***C*** using SOM neurons, subsampled Non-SOM neurons, and best cells in AC and PPC). Dotted line corresponds to chance performance (50%). *N* = 24 AC datasets, *N* = 20 PPC datasets. Error bars indicate the SEM. ****p < 0.0001.

To examine sound location coding at the level of neural populations, we constructed population decoders to predict the most likely sound stimulus location (left vs right) using the activity of different subsets of neurons. Each decoder was based on a Bayesian inversion of an encoding model that related the activity of each neuron to sound location and timing, running behavior, and pupil size (Fig. 3*A*; Materials and Methods, subsection Decoding; Runyan et al., 2017). The posterior probability of each stimulus location was computed cumulatively at each time point using population activity from all previous timepoints in the trial. Decoder performance was quantified as the fraction of trials where the stimulus with the maximal posterior probability matched the actual presented stimulus.

First, to compare the overall ability of AC and PPC to represent sound location, we used activity of only the best cells (i.e., individual neurons whose activity best decoded sound location) regardless of cell type in the population decoder. The number of best cells used for each dataset was chosen to match the number of SOM neurons imaged during that session. The performance of the “best cell” decoder was above chance when using activity from both AC and PPC (Fig. 3*C*,*D*; Materials and Methods, subsection Decoding). However, as expected based on the sound location sensitivity of individual neurons (Fig. 2), AC decoding accuracy was higher than that of PPC (*p* < 0.0001). Next, we compared the SOM and Non-SOM population decoders from each dataset. Within both areas, sound location decoding was similar when using the activity of either SOM or Non-SOM populations (AC, *p* = 0.82; PPC, *p* = 0.56; unpaired permutation tests; Fig. 3*C*), and both cell type populations from AC outperformed decoding based on PPC populations (*p* < 0.0001, unpaired permutation test; Fig. 3*C*,*D*). All cell type-specific, subsampled population decoders performed worse than the decoders based on the *n*-matched population of best neurons (*p* < 0.0001, paired permutation test; Fig. 3*D*; see Materials and Methods, subsection Decoding). To summarize, sound location was more accurately decoded from AC population activity than PPC, and SOM and random *n*-matched Non-SOM subpopulations in both areas were similarly informative about sound location. A sparse code for sound location was especially evident in AC, as small numbers of highly tuned best neurons more accurately encoded sound location than the random subsamples of the population (Table 1, full values and statistics).

**Table 1:** Left-Right (LR) sound location decoding/full values and statistics related to Figures 3 and 6

Group	*n*	Mean	SD	95% Confidence interval of the mean	*p* value
AC best cells – LR^1^	24 × 10	0.86	0.11	0.85–0.87	
AC Non-SOM – LR^2^	24 × 10	0.68	0.13	0.66–0.69	
AC SOM – LR^3^	24 × 10	0.68	0.12	0.66–0.69	
PPC best cells – LR^4^	20 × 10	0.65	0.10	0.64–0.67	
PPC Non-SOM – LR^5^	20 × 10	0.53	0.09	0.52–0.53	
PPC SOM – LR^6^	20 × 10	0.52	0.09	0.51–0.53	
Kruskal–Wallis^1–6^					<0.0001
Unpaired permutation^1,2^					<0.0001
Unpaired permutation^1,3^					<0.0001
Unpaired permutation^1,4^					<0.0001
Unpaired permutation^1,5^					<0.0001
Unpaired permutation^1,6^					<0.0001
Unpaired permutation^2,3^					0.8167
Unpaired permutation^2,4^					0.1189
Unpaired permutation^2,5^					<0.0001
Unpaired permutation^2,6^					<0.0001
Unpaired permutation^3,4^					0.1160
Unpaired permutation^3,5^					<0.0001
Unpaired permutation^3,6^					<0.0001
Unpaired permutation^4,5^					<0.0001
Unpaired permutation^4,6^					<0.0001
Unpaired permutation^5,6^					0.4644
AC high arousal – LR (all cells)	24 × 10	0.83	0.16	0.81–0.85	0.0003
AC low arousal – LR (all cells)	24 × 10	0.78	0.12	0.76–0.80	
PPC high arousal – LR (all cells)	20 × 10	0.54	0.13	0.53–0.55	0.060
PPC low arousal – LR (all cells)	20 × 10	0.57	0.13	0.56–0.59	

The superscripts label the samples being compared in the statistics below.

### Activity of SOM neurons was differently modulated during aroused states in AC and PPC

**Figure 4.**
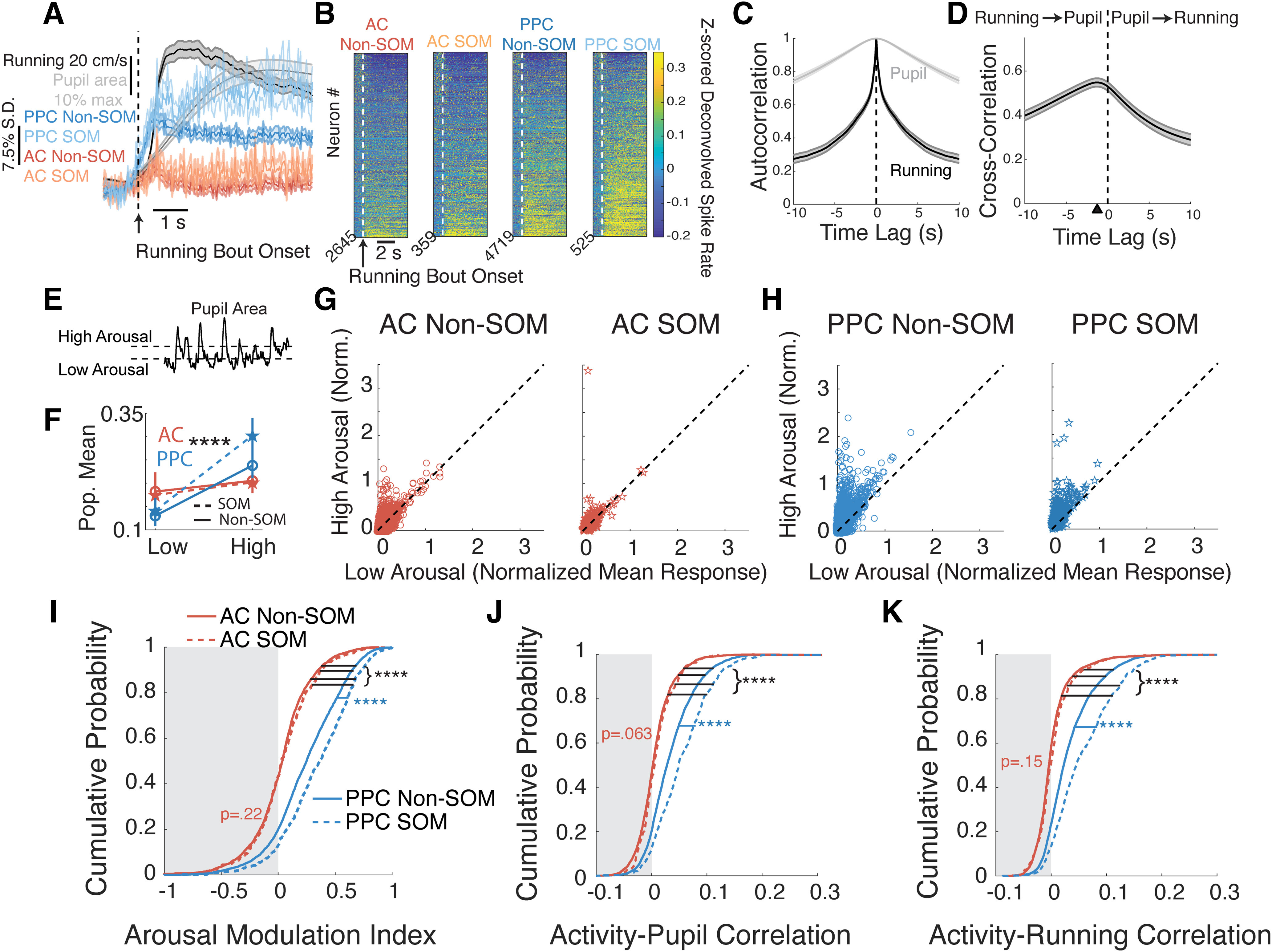
Single-cell activity changes with arousal during the spontaneous context. ***A***, Average *z*-scored deconvolved activity, running speed, and pupil area aligned on running bout onset. Red, The activity of AC Non-SOM neurons (*N* = 24 datasets); orange, AC SOM neuron activity (*N* = 24 datasets); dark blue, PPC Non-SOM neuron activity (*N* = 20 datasets); light blue, PPC SOM neuron activity (*N* = 20 datasets); black, running speed (*N* = 44 datasets); gray, pupil area (*N* = 44 datasets). ***B***, Average *z*-scored deconvolved activity of individual neurons, from left to right: all AC Non-SOM, AC SOM, PPC Non-SOM, and PPC SOM neurons aligned on running bout onset (AC Non-SOM, *N* = 2645; AC SOM, *N* = 359; PPC Non-SOM, *N* = 4719; PPC SOM, *N* = 525; ***B***–***I***). Neurons were sorted by running modulation. ***C***, Autocorrelation of pupil area and running speed, averaged across all datasets (*N* = 44). ***D***, Cross-correlation between running speed and pupil area, averaged across all datasets (*N* = 44). Black triangle indicates the time lag with peak correlation, with running preceding pupil by 1.27 s. ***E***, Illustration of clustering that sorted pupil area during each imaging frame into low, transition, and high arousal states. ***F***, Mean responses of AC Non-SOM (red solid), AC SOM (red dotted), PPC Non-SOM (blue solid), and PPC SOM (blue dotted) neurons in low and high arousal states, as defined in ***E***. ***G***, Left, Mean activity of AC Non-SOM neurons in high arousal state (classified with clustering as in ***E***), plotted against mean activity in the low arousal state. Right, Mean activity of AC SOM neurons in high arousal versus low arousal states. ***H***, As in ***G***, for PPC Non-SOM and SOM neurons. ***I***, Cumulative probability distribution of the arousal modulation index in AC Non-SOM (solid red), AC SOM (dotted red), PPC Non-SOM (solid blue), and PPC SOM (dotted blue) neurons. The arousal modulation index was calculated from the values in ***G*** and ***H***, (High – Low)/(High + Low) for each neuron. ***J***, Cumulative probability distribution of the Pearson correlation between the activity and pupil area of each neuron; colors are as in ***I***. ***K***, Cumulative probability distribution of the Pearson correlation between activity and running speed in each neuron. Significance was determined by permutation test: ****p < 0.0001. Error bars, 95% bootstrapped confidence interval around the mean.

Next, we more thoroughly characterized the activity of SOM and Non-SOM neurons during arousal state transitions. We focused these analyses on the spontaneous context, to isolate the effects of state transitions from the effects of sound stimulation on activity. We defined behavioral state transitions as the onset times of “running bouts,” when the mouse’s running speed rapidly increased (Materials and Methods, subsection Running bouts and modulation). We aligned and averaged the pupil area measurements to running bout onsets, observing that pupil area also increased during running bouts, though with a slower time course (Fig. 4*A*, gray trace; *N* = 44 imaging sessions). We also aligned and averaged the activity of SOM and Non-SOM populations from AC and PPC to running bout onset (Fig. 4*A*, colored traces; AC, *N* = 24 imaging sessions; PPC, *N* = 20 imaging sessions; from six mice). In both AC and PPC, mean activity of SOM and Non-SOM neurons increased with the onset of running bouts. However, this increase in activity was weak in AC because of the prevalence of both positively and negatively modulated SOM and Non-SOM neurons in the AC population (Figs. 1*I*, 4*B*). In PPC, Non-SOM and SOM neurons more uniformly increased activity at running onset (Figs. 1*I*, 4*B*).

Because of the slow time course of changes in pupil area compared with the more rapid transitions in locomotion (Fig. 4*C*,*D*), we also characterized single-neuron activity during periods of sustained pupil constriction and dilation. We classified pupil measurements as corresponding to low, transitional, or high arousal states (Fig. 4*E*; Materials and Methods, subsection Defining low and high arousal states based on pupil area), and focused analyses on the low and high arousal states. As expected from the relationship between running speed and pupil area, running speed was higher during the pupil-defined high arousal states than low arousal states (*p* < 0.0001). Mice ran at a mean ± SEM of 48.24 ± 4.77 cm/s during the high arousal state, and 7.71 ± 1.29 cm/s during the low arousal state (*p* < 0.0001; *N* = 44 datasets). SOM and Non-SOM activity was elevated overall in high arousal states (*p* < 0.0001 for AC Non-SOM, PPC SOM, and Non-SOM; *p* = 0.0029 for AC SOM neurons, paired permutation test; Fig. 4*E–H*). To compare arousal modulation of activity in each population of neurons, we next computed an AMI (Materials and Methods, subsection Arousal modulation index), which could vary from −1 to +1, with −1 indicating greater mean activity in the low arousal period, and +1 indicating greater activity in the high arousal period. The AMI was higher in PPC than AC neurons (*p* < 0.0001; Table 2, full values and statistics). Interestingly, the AMI differed by cell type in PPC but not AC. In AC, the AMI was similar in SOM and Non-SOM neurons (*p* = 0.22), while in PPC, the AMI was higher in SOM than Non-SOM neurons (*p* < 0.0001; Fig. 4*I*).

**Table 2:** Arousal modulation index during spontaneous context/full values and statistics related to Figure 4*I*

Group	*n*	Mean	SD	95% Confidence interval of the mean	*p* value
AC SOM arousal modulation index^1^	359	0.057	0.29	0.027–0.085	
AC Non-SOM, arousal modulation index^2^	2645	0.035	0.28	0.025–0.046	
PPC Non-SOM arousal modulation index^3^	4719	0.25	0.31	0.24–0.26	
PPC SOM arousal modulation index^4^	525	0.35	0.31	0.32–0.37	
Kruskal–Wallis^1–4^					7.2E-12
Unpaired permutation^1,2^					0.22
Unpaired permutation^1,3^					<0.0001
Unpaired permutation^2,3^					<0.0001
Unpaired permutation^1,4^					<0.0001
Unpaired permutation^2,4^					<0.0001
Unpaired permutation^3,4^					<0.0001

Finally, to consider the full-time-varying relationship between ongoing neuronal activity and behavioral correlates of arousal, we correlated the activity of each neuron to pupil area and running speed, across the entire spontaneous behavioral context (Fig. 4*J*,*K*). Consistent with the above analyses based on activity aligned on state transitions, SOM and Non-SOM activity in PPC was more strongly correlated with both running and pupil size than in AC (*p* < 0.0001). Within AC, SOM, and Non-SOM activity was similarly correlated with the two behavioral measures (pupil area, *p* = 0.063; running speed, *p* = 0.146), while within PPC the activity of SOM neurons was again more strongly correlated with behavior than was the activity of Non-SOM neurons (*p* < 0.001 for both running and pupil; Fig. 4*J*,*K*, Tables 3, 4, 5).

**Table 3:** Correlation coefficients with pupil area and running speed during spontaneous context/full values and statistics related to Figure 4, *J* and *K*

Group	*n*	Mean	SD	95% Confidence interval of the mean	*p* value
AC Non-SOM, pupil correlation coefficient^1^	2645	0.0057	0.033	0.0045–0.0070	
AC SOM pupil correlation coefficient^2^	359	0.0091	0.031	0.0061–0.12	
PPC Non-SOM pupil correlation coefficient^3^	4719	0.035	0.043	0.034–0.036	
PPC SOM pupil correlation coefficient^4^	525	0.055	0.049	0.051–0.059	
Kruskal–Wallis^1-4^					6.3E-250
Unpaired permutation^1,2^					0.063
Unpaired permutation^1,3^					<0.0001
Unpaired permutation^2,3^					<0.0001
Unpaired permutation^1,4^					<0.0001
Unpaired permutation^2,4^					<0.0001
Unpaired permutation^3,4^					<0.0001
AC Non-SOM, running speed correlation coefficient^1^	2645	0.0020	0.034	0.00060–0.0033	
AC SOM running speed correlation coefficient^2^	359	0.0048	0.034	0.0013–0.0089	
PPC Non-SOM running speed correlation coefficient^3^	4719	0.033	0.044	0.032–0.034	
PPC SOM running speed correlation coefficient^4^	525	0.057	0.055	0.053–0.062	
Kruskal–Wallis^1–4^					<0.0001
Unpaired permutation^1,2^					0.15
Unpaired permutation^1,3^					<0.0001
Unpaired permutation^2,3^					<0.0001
Unpaired permutation^1,4^					<0.0001
Unpaired permutation^2,4^					<0.0001
Unpaired permutation^3,4^					<0.0001

**Table 4:** Arousal modulation index during passive listening context

Group	*n*	Mean	SD	95% Confidence interval of the mean	*p* value
AC Non-SOM, arousal modulation index^1^	2645	0.072	0.26	0.063–0.083	
AC SOM arousal modulation index^2^	359	0.079	0.28	0.049–0.11	
PPC Non-SOM arousal modulation index^3^	4719	0.18	0.29	0.17–0.19	
PPC SOM arousal modulation index^4^	525	0.26	0.28	0.24–0.29	
Kruskal–Wallis^1–4^					2.1E-66
Unpaired permutation^1,2^					0.63
Unpaired permutation^1,3^					<0.0001
Unpaired permutation^2,3^					<0.0001
Unpaired permutation^1,4^					<0.0001
Unpaired permutation^2,4^					<0.0001
Unpaired permutation^3,4^					<0.0001

**Table 5:** Correlation coefficients with pupil area and running speed during passive listening context

Group	*n*	Mean	SD	95% Confidence interval of the mean	*p* value
AC Non-SOM, pupil correlation coefficient^1^	2645	0.0096	0.031	0.0081–0.010	
AC SOM pupil correlation coefficient^2^	359	0.010	0.033	0.0074–0.014	
PPC Non-SOM pupil correlation coefficient^3^	4719	0.030	0.042	0.029–0.031	
PPC SOM pupil correlation coefficient^4^	525	0.043	0.044	0.038–0.046	
Kruskal–Wallis^1-4^					5.3E-115
Unpaired permutation^1,2^					0.63
Unpaired permutation^1,3^					<0.0001
Unpaired permutation^2,3^					<0.0001
Unpaired permutation^1,4^					<0.0001
Unpaired permutation^2,4^					<0.0001
Unpaired permutation^3,4^					<0.0001
AC Non-SOM, running speed correlation coefficient^1^	2645	0.0067	0.029	0.0056–0.0078	
AC SOM running speed correlation coefficient^2^	359	0.0076	0.30	0.0050–0.011	
PPC Non-SOM running speed correlation coefficient^3^	4719	0.029	0.042	0.028–0.030	
PPC SOM running speed correlation coefficient^4^	525	0.045	0.047	0.038–0.046	
Kruskal–Wallis^1–4^					1.2E-146
Unpaired permutation^1,2^					0.59
Unpaired permutation^1,3^					<0.0001
Unpaired permutation^2,3^					<0.0001
Unpaired permutation^1,4^					<0.0001
Unpaired permutation^2,4^					<0.0001
Unpaired permutation^3,4^					<0.0001

Together, our results so far indicate that in the absence of sound stimulation, SOM and Non-SOM neurons have heterogeneous activity relationships with arousal state in AC, whether defined by running speed or pupil area. In PPC, neuronal activity was positively modulated with heightened arousal, with a stronger modulation of SOM neurons than Non-SOM neurons.

### An encoding model revealed different contributions of running speed and pupil size to single cell activity in AC and PPC

Running speed and pupil area are strongly correlated signals but vary on different timescales (Fig. 4*C*,*D*) and have separable effects on neuronal activity (Vinck et al., 2015). Our analyses of ongoing spontaneous activity in AC and PPC also hint at the possibility of separable effects of pupil area and running speed, as the activity of AC Non-SOM neurons was more highly correlated with pupil area than with running speed (*p* < 0.0001, paired permutation test).

**Figure 5.**
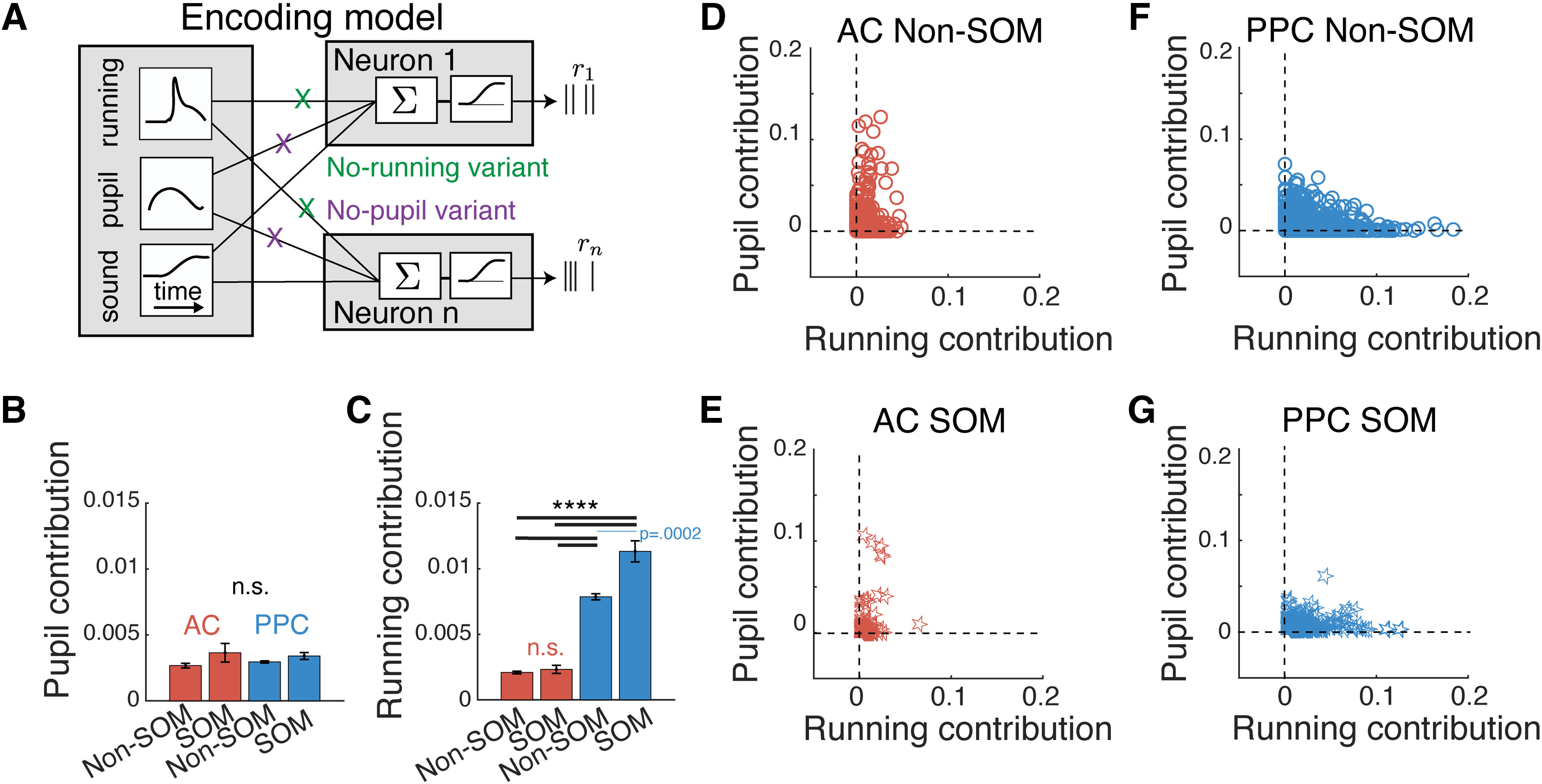
Using an encoding model to disentangle running and pupil contributions to single-cell activity. ***A***, Schematic of GLM used to determine the unique contributions of pupil area and running velocity to neuron activity. ***B***, Average contribution of pupil area to activity across all AC Non-SOM (*N* = 2645) and SOM (*N* = 359) neurons (red bars) and PPC Non-SOM (*N* = 4525) and SOM (*N* = 505) neurons (blue bars). Contribution was quantified as the improvement in model prediction performance of the activity of each neuron when adding pupil area as a set of predictors. Error bars indicate the SEM. n.s., Not significant. Exact values are in the text. ***C***, As in ***B***, calculated when comparing model performance with and without running velocity predictors. AC Non-SOM and SOM neurons (red bars) and PPC Non-SOM and SOM neurons (blue bars), ****p < .0001. ***D***, Pupil contribution plotted against running contribution for each AC Non-SOM neuron, calculated as in ***B***. ***E***, Pupil contribution plotted against running contribution for each AC SOM neuron. ***F***, ***G***, Pupil contribution plotted against running contribution for each PPC Non-SOM or SOM neuron. Sample sizes in ***B*** apply to all panels.

To disentangle the relationships among neuronal activity, running velocity, and pupil area, we used an encoding model approach (Pillow et al., 2008; Park et al., 2014; Runyan et al., 2017). We constructed a GLM that used sound stimulus timing and location, pupil area, and running velocity to predict the responses of individual SOM and Non-SOM neurons in AC and PPC, in the passive listening context (Fig. 5*A*). We note that in the above analyses (Figs. 1, 4), we considered only the speed at which the mouse was running in any direction, as increased running speed is correlated with heightened arousal (Fu et al., 2014; Zhou et al., 2014; Vinck et al., 2015; Mineault et al., 2016; Shimaoka et al., 2018). Because the activity of PPC neurons can be selective for running direction (Nitz, 2006; Whitlock et al., 2012; Runyan et al., 2017; Krumin et al., 2018; Minderer et al., 2019) in the GLM, we used running velocity rather than speed to obtain more accurate predictions of the activity of each neuron (Materials and Methods, subsection Encoding models; Fig. 5).

To determine the relative contributions of pupil area and running velocity to the activity of neurons, we separately removed these predictors from the model and measured the decrement in the prediction performance of the model. For example, we calculated the pupil size contribution to the activity of a given neuron as the difference between the prediction performance of the model with and without the pupil size predictors. We considered this decrement in model performance as the contribution of pupil size to the activity of the neuron that is not redundant with running speed (Materials and Methods, subsection Running and pupil contribution). If running velocity and pupil area did not make unique contributions to neuronal activity, running predictors would be able to account for the missing pupil area predictors and vice versa, and the performance of the model would not be degraded compared with the full model, which includes both pupil area and running velocity predictors. The model comparison revealed single-neuron activity that could be explained distinctly by running and by pupil in both AC and PPC [Fig. 5*B–G* (note neurons along the pupil and running contribution axes in *D–G*)]. While a Kruskal–Wallis test indicated that the contribution of pupil size differed among the four cell type/area combinations, *post hoc* tests returned *p* > 0.05 for all pairwise comparisons (Table 6, full values and statistics). In contrast, running contributions were overall stronger in PPC neurons than in AC neurons (*p* < 0.0001) and within PPC, running contributions were stronger to SOM than Non-SOM activity (*p* = 0.0002; Fig. 5*C*,*F*,*G*). Within AC, running contributions did not depend on cell type (*p* = 0.44; Fig. 5*C*,*D*,*E*; Table 6, full values and statistics). Thus, running contributions, but not pupil contributions, reflect the observed differences in the arousal dependence of activity in AC and PPC.

**Table 6:** Pupil and running contributions/full values and statistics related to **Figure 5**

Group	*n*	Mean	SD	95% Confidence interval of the mean	*p* value
AC Non-SOM, pupil diameter contribution^1^	2476	0.0027	0.0086	0.0023–0.0031	
AC SOM, pupil diameter contribution^2^	330	0.0036	0.0123	0.0025–0.0049	
PPC Non-SOM, pupil diameter contribution^3^	4525	0.0029	0.0060	0.0028–0.0031	
PPC SOM, pupil diameter contribution^4^	505	0.0034	0.0059	0.0029–0.0040	
Kruskal–Wallis^1-4^					<0.0001
Unpaired permutation^1,2^					0.0780
Unpaired permutation^1,3^					0.1259
Unpaired permutation^2,3^					0.2360
Unpaired permutation^1,4^					0.0619
Unpaired permutation^2,4^					0.9520
Unpaired permutation^3,4^					0.1070
AC Non-SOM, running speed contribution^1^	2476	0.0020	0.0050	0.0019–0.0023	
AC SOM running speed contribution^2^	330	0.0023	0.0005	0.0019-0.0320	
PPC Non-SOM running speed contribution^3^	4525	0.0079	0.0156	0.0075–0.0083	
PPC SOM running speed contribution^4^	505	0.0113	0.0181	0.0099–0.0131	
Kruskal–Wallis^1-4^					<0.0001
Unpaired permutation^1,2^					0.4409
Unpaired permutation^1,3^					0.0001
Unpaired permutation^2,3^					0.0001
Unpaired permutation^1,4^					0.0001
Unpaired permutation^2,4^					0.0001
Unpaired permutation^3,4^					0.0002

### Sound location coding was enhanced with arousal in AC, but not PPC

**Figure 6.**
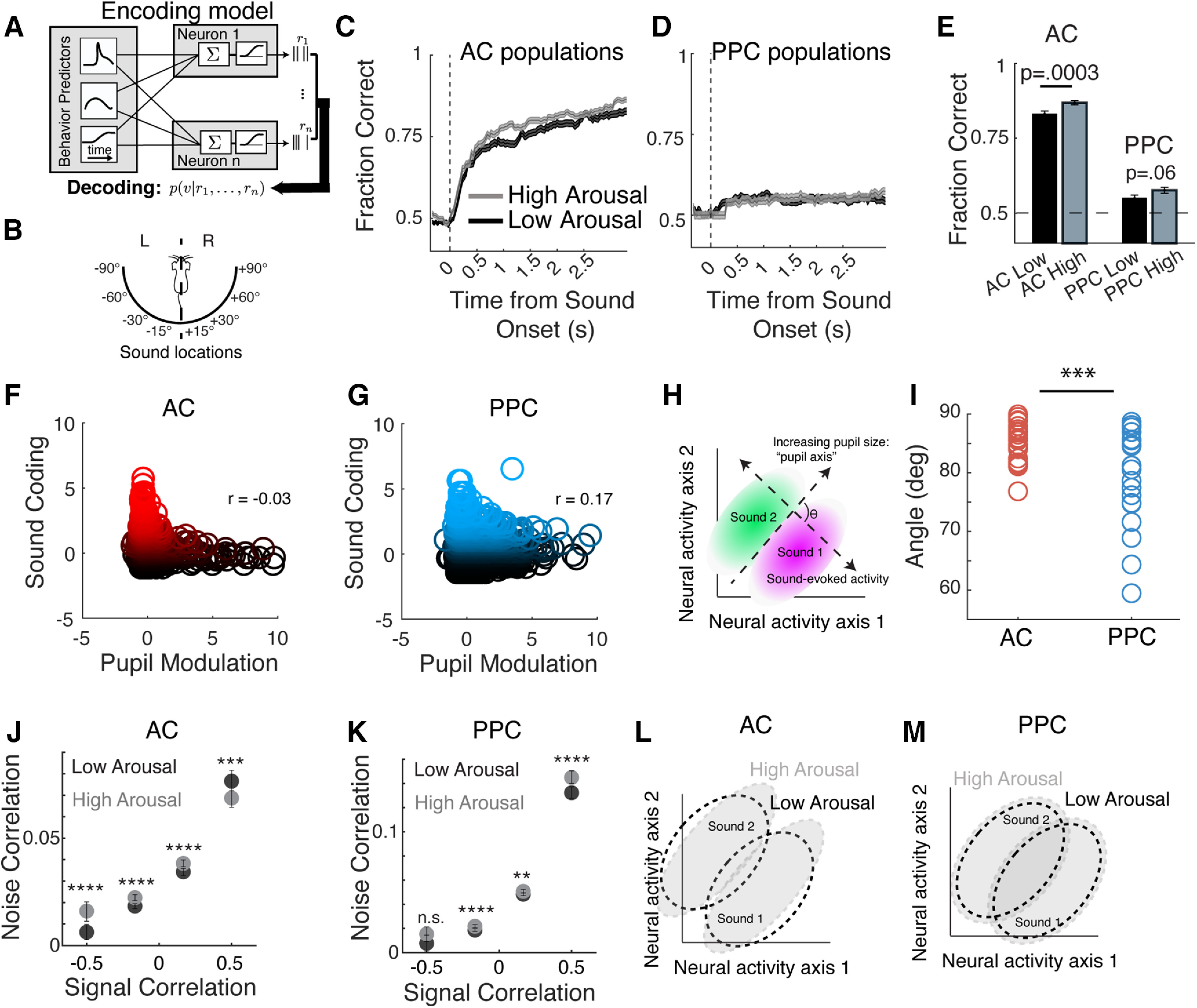
Arousal impacts the structure of population activity, improving information coding in AC. ***A***, The encoding model was trained on all trials that included all arousal levels, and inverted using Bayes’ rule to compute the posterior probability of auditory stimuli given the activity of the neural population in AC and PPC. ***B***, Schematic of the discrimination being performed by the decoder, classifying sound stimuli as occurring from the left or right of the mouse. ***C-D***, Mean fraction correct of cumulative sound location (left sound locations vs right sound locations) decoding in AC (***C***) and PPC (***D***) populations in high (dark) and low (light) arousal conditions. Subsampling to match arousal and sound locations was repeated 10 times. ***E***, Cumulative fraction correct of decoding for left versus right sound location at the end of the trial in AC and PPC in high and low arousal. ***F***, For each AC neuron, the sound location decoding performance based on the activity of that neuron is plotted against the pupil modulation of that neuron, as quantified by the encoding model in Figure 5. Neurons are color coded from black to red to indicate their *z*-scored sound location decoding performance (red neurons > 0 *z*-scored performance). ***G***, As in ***F***, for PPC. ***H***, Schematic to explain the definitions of the sound location and pupil axes in the high-dimensional population activity space in ***I***. ***I***, Angle in degrees between the pupil and sound location axes in each AC and PPC dataset. The angle between the pupil and sound axes is significantly smaller in PPC than AC. ***J***, Mean pairwise noise correlation among sound-responsive AC neurons during high arousal (gray) and low arousal (black) trials, binned by their pairwise signal correlation. ***K***, As in ***J***, but for PPC. ***L***, Schematic demonstrating the impacts of arousal on sound coding in AC population activity. The shape of the population activity subspace responding to “sound 1” and “sound 2” changes between low arousal (dashed outlines) and high arousal (gray ovals) states, improving their discriminability. This shape change occurs because the shared variability is reduced among neurons coding for similar locations and is enhanced among neurons coding for different locations (see ***J***). ***M***, As in ***L***, for PPC. In PPC, the shape of the area of population activity space encoding sound 1 or sound 2 does not change with arousal, as shared variability is more generally increased across neuron pairs (see ***K***). Across panels: **p < 0.01, ***p < 0.001, ****p < 0.0001. n.s., Not significant. AC Non-SOM, *N* = 24 datasets; AC SOM, *N* = 24 datasets; PPC Non-SOM, *N* = 20 datasets; PPC SOM, *N* = 20 datasets; throughout figure panels.

Given the activity changes with arousal in AC and PPC, we hypothesized that arousal would affect sensory information coding in both regions. To test this possibility, we trained and tested the sound location decoder (Fig. 3) in low and high arousal periods (Fig. 6*A–E*). Sound stimulus trials were classified as occurring during low or high arousal states based on pupil size (Fig. 4*E*), and low and high arousal trials were evenly balanced to train and test the decoders. Decoding performance for left versus right sound locations using AC populations was modestly improved during heightened arousal (*p* = 0.0003, paired permutation test), but decoding performance was similarly poor in low and high arousal trials using PPC population activity (Fig. 6*C–E*; *p* = 0.06, paired permutation test). Thus, while sensory coding in AC was slightly improved with heightened arousal, coding within PPC populations was unaffected by arousal state (Table 1, full values and statistics).

To better understand the basis for the improvement in sound location coding with arousal in AC, we related the sound coding of individual neurons (*z*-scored decoding performance) to the contribution of pupil size to their activity (Fig. 5, encoding model). In AC, sound coding had a weakly negative relationship with pupil modulation (Pearson’s correlation, −0.03), where the neurons with the strongest sound coding tended to have the weakest pupil modulation. In PPC, on the other hand, sound coding and pupil modulation were positively correlated (Pearson’s correlation, 0.17), as the same neurons can be modulated by both sound and arousal state in PPC. We then examined the modulation of neural population activity by sounds and by arousal state, in population activity space, where each dimension (axis) is the activity of one neuron and a population of *n* neurons has *n* dimensions (axes; Cunningham and Yu, 2014). We found the direction of the “sound location axis,” along which left versus right sound locations were best distinguished in this *n*-dimensional space, and the direction of the “pupil axis,” along which pupil size best explained population activity, in each AC and PPC population (see Materials and Methods, subsection Defining population activity axes related to sound location and arousal). We then measured the angle between these axes. In AC, these angles were more orthogonal to each other than in PPC (*p* = 0.0006).

The correlation structure of population activity influences the amount of information that can be encoded across neurons (Averbeck et al., 2006; Panzeri et al., 2022). Specifically, the slope of the relationship between signal and noise correlations determines whether noise correlations limit the information that can be encoded by the population. In other words, greater shared variability among neurons with similar tuning preferences reduces the information that the population can encode. As a consequence, reductions in noise correlations among neuron pairs with similar tuning preferences or increases in noise correlations among neuron pairs with opposing tuning preferences would both theoretically increase the encoding capacity of a neural population (Averbeck et al., 2006; Panzeri et al., 2022). We then wondered whether transitions between arousal states also induced different changes in the correlation structure in AC and PPC, contributing to the improvements in sound location coding in AC. We compared pairwise noise correlations in high and low arousal conditions for both regions, and sorted these by the signal correlations between neuron pairs. Pairwise noise correlations among neurons with similar sound location preferences (high signal correlations) were reduced in the high arousal state in AC (*p* = 0.002). In PPC, on the other hand, these similarly tuned neurons instead became more correlated with arousal (*p* < 0.0001). In neuron pairs with negative signal correlations (different tuning), noise correlations were enhanced in AC (*p* = 0.0015), but did not change with arousal in PPC (*p* = 0.235). Together, the arousal-induced changes in the correlation structure of population activity suggest that the improvement in sound location coding in AC could result from a reduction in shared variability across neurons with similar sound preferences, in addition to the generalized suppression in sound-evoked responses during locomotion (Schneider et al., 2014; Bigelow et al., 2019; Yavorska and Wehr, 2021) that could sharpen response selectivity.

## Discussion

Our goal in this study was to determine whether the activity of inhibitory interneurons is differently modulated by behavioral state across the cortical processing hierarchy. We have measured spontaneous and sound-driven activity in populations of SOM-expressing inhibitory interneurons and Non-SOM neurons in layer 2/3 of AC and in PPC, while mice transitioned between arousal states.

In the absence of sound stimulation, the effects of arousal state on spontaneous activity in AC were complex. Although heightened arousal had a slight positive effect on activity in AC at the population level, activity in individual AC neurons could be positively or negatively modulated, as has been described previously by others (Bigelow et al., 2019; Yavorska and Wehr, 2021). Short spurts of running, as we observed in our imaging sessions (Extended Data Fig. 1-1*D*), are associated with a net depolarization in AC (Shimaoka et al., 2018), which likely contributes to the net positive relationship between spontaneous AC activity and running observed here. In contrast, the spontaneous activity of most PPC neurons increased during these heightened arousal states, and SOM neurons were even more strongly and uniformly modulated than Non-SOM neurons.

The behavioral state transitions in our study involved increases both in pupil size and in locomotion (Fig. 4*A*). It is thus crucial to emphasize that the effects of heightened arousal state on neural activity described here include a mixture of motor-related and “true” arousal-related effects, such as those resulting from increases in norepinephrine and acetylcholine release within AC and PPC. While locomotion, whisking, and pupil dilations have all been considered as behavioral correlates of the arousal state of an animal, motor-related feedback acts on specialized circuits within visual, somatosensory, and auditory cortices, determining whether motor behavior positively or negatively influences neural activity (Lee et al., 2013; Fu et al., 2014; Schneider et al., 2014; Pakan et al., 2016; Bigelow et al., 2019; Yavorska and Wehr, 2021). This motor-related feedback acts in concert with neuromodulatory inputs more directly related to the arousal state of the animal, leading to state-dependent network changes that are specialized across cortical regions. It is unlikely that any aroused state is without a related change in fidgeting, facial movements, or other motor outputs (Musall et al., 2019; Stringer et al., 2019), and so the relative contributions of motor-related versus neuromodulatory inputs to neural activity must be defined for each brain region for a full consideration of state-dependent processing across the cortex. Here, we were able to disentangle the unique contributions of running velocity and pupil size to the activity of individual neurons in AC and PPC using an encoding model (Fig. 5). In both regions, pupil size and running velocity had distinguishable contributions to the activity of SOM and Non-SOM neurons. The magnitude of unique pupil contributions was similar across all groups, but running contributions to activity were significantly stronger in PPC, particularly among SOM neurons.

In AC, the effects of arousal and locomotion can oppose each other, as motor-related feedback activates PV neurons, reducing sound-evoked responses during locomotion (Schneider et al., 2014; Bigelow et al., 2019; Yavorska and Wehr, 2021). We observed a small number of Non-SOM neurons in AC that were strongly and positively modulated with locomotion, which may include the PV neurons mediating the locomotion-related reduction of activity in other neurons (Schneider et al., 2014). In PPC, motor-related effects were largely positive and were particularly strong among SOM neurons, suggesting that, unlike in AC (Schneider et al., 2014), motor feedback does not target PV neurons in PPC. Instead, SOM neurons in PPC may inhibit PV neurons during locomotion, disinhibiting excitatory neurons and further enhancing the arousal-related increase in activity in the local population. It will be interesting to determine whether this is the case in future studies. Recently, we also discovered that activity within the SOM population is highly coordinated, especially in PPC (Khoury et al., 2022). As a result, the transitions from low to high arousal states would trigger highly coordinated SOM population events, which could strongly impact the local network activity state (Chen et al., 2015; Veit et al., 2017; Wang and Yang, 2018). In the future, causal manipulations of SOM neurons, mimicking their activity during arousal transitions, will help reveal the impact of these coordinated SOM activity events on the activity and coding in the local population.

The effects of arousal on stimulus coding have been examined in depth across primary sensory cortices (Zhou et al., 2014; McGinley et al., 2015; Vinck et al., 2015; Shimaoka et al., 2018; Lin et al., 2019). Previous studies revealed that moderate levels of arousal optimally impact sensory coding (McGinley et al., 2015; Lin et al., 2019), aligning well with the Yerkes–Dodson (inverted-U) relationship between arousal and perceptual task performance (Waschke et al., 2019). Unlike these studies, we did not observe a decrement in sound location coding in the highest arousal state, instead measuring a modest improvement in decoding accuracy in the high arousal state. Mice in our experiments were most likely to visit two separable behavioral states (stationary/unaroused or running/aroused), without the gradation of different levels of arousal observed by others. As a result, the high arousal state described here likely includes a mixture of the moderate and high arousal states defined by others (McGinley et al., 2015). Because sensory coding in PPC depends on the behavioral relevance of stimuli (Fitzgerald et al., 2011; Pho et al., 2018), we expected that sound coding would also improve in PPC with heightened arousal, when mice might be more aware of the sound stimuli. Surprisingly, despite the more pronounced positive modulation of activity in PPC that accompanied increases in arousal and locomotion (Fig. 4), the behavioral state did not affect sensory encoding in PPC (Fig. 6). Our results imply that arousal alone is not sufficient to improve sensory coding in PPC outside of a task context, supporting the idea that task engagement and arousal modulate sensory responses through separate pathways (Saderi et al., 2021).

Finally, to better understand the basis of the improvement of sound coding with arousal in AC (and lack thereof in PPC), we related sound coding and pupil-related effects on population activity and its correlation structure. In AC, sound coding and pupil modulation were strongest in distinct sets of neurons (Fig. 6*F*), and, with heightened arousal, shared variability was reduced among neurons with similar tuning (Fig. 6*J*). The net result was an improvement in our ability to decode sound information from AC population activity in the heightened arousal state, as the responses of the population to different stimuli were more separable (Fig. 6*L*, scheme). In PPC, on the other hand, sound coding and pupil modulation were intermixed within the same neurons (Fig. 6*G*), and shared variability increased among neurons with similar tuning (Fig. 6*K*). As a consequence, although activity was strongly modulated in PPC with arousal, the separability of population responses to different sounds was not affected (Fig. 6*M*). As has been recently reviewed, positive noise correlations among neurons with similar tuning limit the information that a population can encode (Averbeck et al., 2006; Panzeri et al., 2022), which is consistent with our results. To summarize, arousal had different effects on the correlation structure of population activity in AC and PPC. In AC, the result was better separability of the population responses to different sounds, while in PPC the effects of arousal on population activity were information limiting (Averbeck et al., 2006; Panzeri et al., 2022).

To conclude, we have characterized the effects of the global arousal state on population activity in sensory and association cortices by measuring neuronal activity during fluctuations in arousal and locomotion. In AC, but not PPC, sensory representations were enhanced with arousal, even when not behaviorally relevant. An important future direction will be to determine whether global shifts in arousal affect the coding of behaviorally relevant information in PPC, and whether local inhibitory circuits can provide a gating mechanism to enhance the encoding of behaviorally relevant sensory information of PPC.

## Synthesis

Reviewing Editor: David Schoppik, New York University Decisions are customarily a result of the Reviewing Editor and the peer reviewers coming together and discussing their recommendations until a consensus is reached. When revisions are invited, a fact-based synthesis statement explaining their decision and outlining what is needed to prepare a revision will be listed below. The following reviewer(s) agreed to reveal their identity: NONE. Note: If this manuscript was transferred from JNeurosci and a decision was made to accept the manuscript without peer review, a brief statement to this effect will instead be what is listed below.

I read the revised manuscript and I think that the rebuttal adequately addresses the reviewers confusions and concerns. I think the novel aspects of the study and the contributions to the literature are now sufficiently clear.

To the point about color choice: I defer to the authors, but I will point out that the red and orange render similarly (to my eyes) on screen and when printed out on paper. I recommend that the authors choose colors from either the Ito or Tol palettes of color-blind-friendly hues: https://thenode.biologists.com/data-visualization-with-flying-colors/research/ for an overview. You’ve got pink and minty green available. Your call. I’m making the decision “revise and re-review” so that the system gives you the opportunity to make that change formally.

## References

[B1] Adesnik H, Bruns W, Taniguchi H, Huang ZJ, Scanziani M (2012) A neural circuit for spatial summation in visual cortex. Nature 490:226–231. 10.1038/nature11526 23060193 PMC3621107

[B2] Averbeck BB, Latham PE, Pouget A (2006) Neural correlations, population coding and computation. Nat Rev Neurosci 7:358–366. 10.1038/nrn1888 16760916

[B3] Beierlein M, Gibson JR, Connors BW (2000) A network of electrically coupled interneurons drives synchronized inhibition in neocortex. Nat Neurosci 3:904–910. 10.1038/78809 10966621

[B4] Bennett C, Arroyo S, Hestrin S (2013) Subthreshold mechanisms underlying state-dependent modulation of visual responses. Neuron 80:350–357. 10.1016/j.neuron.2013.08.007 24139040 PMC3806653

[B5] Bigelow J, Morrill RJ, Dekloe J, Hasenstaub AR (2019) Movement and VIP interneuron activation differentially modulate encoding in mouse auditory cortex. Eneuro 6:ENEURO.0164-19.2019. 10.1523/ENEURO.0164-19.2019 PMC6751373 31481397

[B6] Cantero JL, Atienza M, Salas RM, Gómez CM (1999) Brain spatial microstates of human spontaneous alpha activity in relaxed wakefulness, drowsiness period, and REM sleep. Brain Topogr 11:257–263. 10.1023/a:1022213302688 10449257

[B7] Chen N, Sugihara H, Sur M (2015) An acetylcholine-activated microcircuit drives temporal dynamics of cortical activity. Nat Neurosci 18:892–902. 10.1038/nn.4002 25915477 PMC4446146

[B8] Chen T-W, Wardill TJ, Sun Y, Pulver SR, Renninger SL, Baohan A, Schreiter ER, Kerr RA, Orger MB, Jayaraman V, Looger LL, Svoboda K, Kim DS (2013) Ultrasensitive fluorescent proteins for imaging neuronal activity. Nature 499:295–300. 10.1038/nature12354 23868258 PMC3777791

[B9] Cohen MR, Maunsell JHR (2009) Attention improves performance primarily by reducing interneuronal correlations. Nat Neurosci 12:1594–1600. 10.1038/nn.2439 19915566 PMC2820564

[B10] Cohen MR, Maunsell JHR (2010) A neuronal population measure of attention predicts behavioral performance on individual trials. J Neurosci 30:15241–15253. 10.1523/JNEUROSCI.2171-10.2010 21068329 PMC3045704

[B11] Cowley BR, Snyder AC, Acar K, Williamson RC, Yu BM, Smith MA (2020) Slow drift of neural activity as a signature of impulsivity in macaque visual and prefrontal cortex. Neuron 108:551–567.e8. 10.1016/j.neuron.2020.07.021 32810433 PMC7822647

[B12] Cunningham JP, Yu BM (2014) Dimensionality reduction for large-scale neural recordings. Nat Neurosci 17:1500–1509. 10.1038/nn.3776 25151264 PMC4433019

[B13] Dadarlat MC, Stryker MP (2017) Locomotion enhances neural encoding of visual stimuli in mouse V1. J Neurosci 37:3764–3775. 10.1523/JNEUROSCI.2728-16.2017 28264980 PMC5394894

[B14] Dienel SJ, Ciesielski AJ, Bazmi HH, Profozich EA, Fish KN, Lewis DA (2021) Distinct laminar and cellular patterns of GABA neuron transcript expression in monkey prefrontal and visual cortices. Cereb Cortex 31:2345–2363. 10.1093/cercor/bhaa341 33338196 PMC8023857

[B15] Dipoppa M, Ranson A, Krumin M, Pachitariu M, Carandini M, Harris KD (2018) Vision and locomotion shape the interactions between neuron types in mouse visual cortex. Neuron 98:602–615.e8. 10.1016/j.neuron.2018.03.037 29656873 PMC5946730

[B16] Elhilali M, Fritz JB, Klein DJ, Simon JZ, Shamma SA (2004) Dynamics of precise spike timing in primary auditory cortex. J Neurosci 24:1159–1172. 10.1523/JNEUROSCI.3825-03.2004 14762134 PMC6793586

[B17] Fanselow EE, Richardson KA, Connors BW (2008) Selective, state-dependent activation of somatostatin-expressing inhibitory interneurons in mouse neocortex. J Neurophysiol 100:2640–2652. 10.1152/jn.90691.2008 18799598 PMC2585405

[B18] Fino E, Yuste R (2011) Dense inhibitory connectivity in neocortex. Neuron 69:1188–1203. 10.1016/j.neuron.2011.02.025 21435562 PMC3086675

[B19] Fitzgerald JK, Freedman DJ, Assad JA (2011) Generalized associative representations in parietal cortex. Nat Neurosci 14:1075–1079. 10.1038/nn.2878 21765425 PMC3145031

[B20] Friedman J, Hastie T, Tibshirani R (2010) Regularization paths for generalized linear models via coordinate descent. J Stat Soft 33:1–22. 10.18637/jss.v033.i01 PMC2929880 20808728

[B21] Friedrich J, Zhou P, Paninski L (2017) Fast online deconvolution of calcium imaging data. PLoS Comput Biol 13:e1005423. 10.1371/journal.pcbi.1005423 28291787 PMC5370160

[B22] Fu Y, Tucciarone JM, Espinosa JS, Sheng N, Darcy DP, Nicoll RA, Huang ZJ, Stryker MP (2014) A cortical circuit for gain control by behavioral state. Cell 156:1139–1152. 10.1016/j.cell.2014.01.050 24630718 PMC4041382

[B23] Garcia-Junco-Clemente P, Tring E, Ringach DL, Trachtenberg JT (2019) State-dependent subnetworks of parvalbumin-expressing interneurons in neocortex. Cell Rep 26:2282–2288.e3. 10.1016/j.celrep.2019.02.005 30811979 PMC6407626

[B24] Goard M, Dan Y (2009) Basal forebrain activation enhances cortical coding of natural scenes. Nat Neurosci 12:1444–1449. 10.1038/nn.2402 19801988 PMC3576925

[B25] Gould IC, Rushworth MF, Nobre AC (2011) Indexing the graded allocation of visuospatial attention using anticipatory alpha oscillations. J Neurophysiol 105:1318–1326. 10.1152/jn.00653.2010 21228304 PMC3074422

[B26] Grent-’t-Jong T, Boehler CN, Kenemans JL, Woldorff MG (2011) Differential functional roles of slow-wave and oscillatory-α activity in visual sensory cortex during anticipatory visual-spatial attention. Cereb Cortex 21:2204–2216. 10.1093/cercor/bhq279 21372123 PMC3169654

[B27] Harvey CD, Coen P, Tank DW (2012) Choice-specific sequences in parietal cortex during a virtual-navigation decision task. Nature 484:62–68. 10.1038/nature10918 22419153 PMC3321074

[B28] Hromádka T, Deweese MR, Zador AM (2008) Sparse representation of sounds in the unanesthetized auditory cortex. PLoS Biol 6:e16. 10.1371/journal.pbio.0060016 18232737 PMC2214813

[B29] Karnani MM, Jackson J, Ayzenshtat I, Sichani AH, Manoocheri K, Kim S, Yuste R (2016) Opening holes in the blanket of inhibition: localized lateral disinhibition by VIP interneurons. J Neurosci 36:3471–3480. 10.1523/JNEUROSCI.3646-15.2016 27013676 PMC4804006

[B30] Kato HK, Gillet SN, Isaacson JS (2015) Flexible sensory representations in auditory cortex driven by behavioral relevance. Neuron 88:1027–1039. 10.1016/j.neuron.2015.10.024 26586181 PMC4670799

[B31] Kawaguchi Y, Shindou T (1998) Noradrenergic excitation and inhibition of GABAergic cell types in rat frontal cortex. J Neurosci 18:6963–6976. 10.1523/JNEUROSCI.18-17-06963.1998 9712665 PMC6792977

[B83] Khan AG et al. (2018) Distinct learning-induced changes in stimulus selectivity and interactions of GABAergic interneuron classes in visual cortex. Nature Neuroscience 21:851–859. 29786081 10.1038/s41593-018-0143-z PMC6390950

[B32] Khoury CF, Fala NG, Runyan CA (2022) The spatial scale of somatostatin subnetworks increases from sensory to association cortex. Cell Rep 40:111319. 10.1016/j.celrep.2022.111319 36070697 PMC9469807

[B33] Kim Y, Yang GR, Pradhan K, Venkataraju KU, Bota M, del Molino LCG, Fitzgerald G, Ram K, He M, Levine JM, Mitra P, Huang ZJ, Wang X-J, Osten P (2017) Brain-wide maps reveal stereotyped cell-type-based cortical architecture and subcortical sexual dimorphism. Cell 171:456–469.e22. 10.1016/j.cell.2017.09.020 28985566 PMC5870827

[B34] Krumin M, Lee JJ, Harris KD, Carandini M (2018) Decision and navigation in mouse parietal cortex. Elife 7:e42583. 10.7554/eLife.42583 30468146 PMC6300355

[B35] Kuchibhotla KV, Gill JV, Lindsay GW, Papadoyannis ES, Field RE, Sten TAH, Miller KD, Froemke RC (2017) Parallel processing by cortical inhibition enables context-dependent behavior. Nat Neurosci 20:62–71. 10.1038/nn.4436 27798631 PMC5191967

[B36] Lee S, Kruglikov I, Huang ZJ, Fishell G, Rudy B (2013) A disinhibitory circuit mediates motor integration in the somatosensory cortex. Nat Neurosci 16:1662–1670. 10.1038/nn.3544 24097044 PMC4100076

[B37] Licata AM, Kaufman MT, Raposo D, Ryan MB, Sheppard JP, Churchland AK (2017) Posterior parietal cortex guides visual decisions in rats. J Neurosci 37:4954–4966. 10.1523/JNEUROSCI.0105-17.2017 28408414 PMC5426183

[B38] Lin P-A, Asinof SK, Edwards NJ, Isaacson JS (2019) Arousal regulates frequency tuning in primary auditory cortex. Proc Natl Acad Sci U S A 116:25304–25310. 10.1073/pnas.1911383116 31757852 PMC6911239

[B39] Livingstone MS, Hubel DH (1981) Effects of sleep and arousal on the processing of visual information in the cat. Nature 291:554–561. 10.1038/291554a0 6165893

[B40] Madisen L, Zwingman TA, Sunkin SM, Oh SW, Zariwala HA, Gu H, Ng LL, Palmiter RD, Hawrylycz MJ, Jones AR, Lein ES, Zeng H (2010) A robust and high-throughput Cre reporting and characterization system for the whole mouse brain. Nat Neurosci 13:133–140. 10.1038/nn.2467 20023653 PMC2840225

[B41] Marrocco RT, Witte EA, Davidson MC (1994) Arousal systems. Curr Opin Neurobiol 4:166–170. 10.1016/0959-4388(94)90067-1 7913640

[B42] Mayo JP, Cohen MR, Maunsell JHR (2015) A refined neuronal population measure of visual attention. PLoS One 10:e0136570. 10.1371/journal.pone.0136570 26296083 PMC4546609

[B43] McGinley MJ, David SV, McCormick DA (2015) Cortical membrane potential signature of optimal states for sensory signal detection. Neuron 87:179–192. 10.1016/j.neuron.2015.05.038 26074005 PMC4631312

[B44] Minderer M, Brown KD, Harvey CD (2019) The spatial structure of neural encoding in mouse posterior cortex during navigation. Neuron 102:232–248.e11. 10.1016/j.neuron.2019.01.029 30772081 PMC6642748

[B45] Mineault PJ, Tring E, Trachtenberg JT, Ringach DL (2016) Enhanced spatial resolution during locomotion and heightened attention in mouse primary visual cortex. J Neurosci 36:6382–6392. 10.1523/JNEUROSCI.0430-16.2016 27307228 PMC4909779

[B46] Morcos AS, Harvey CD (2016) History-dependent variability in population dynamics during evidence accumulation in cortex. Nat Neurosci 19:1672–1681. 10.1038/nn.4403 27694990 PMC5127723

[B47] Musall S, Kaufman MT, Juavinett AL, Gluf S, Churchland AK (2019) Single-trial neural dynamics are dominated by richly varied movements. Nat Neurosci 22:1677–1686. 10.1038/s41593-019-0502-4 31551604 PMC6768091

[B82] Nakamura K (1999) Auditory spatial discriminatory and mnemonic neurons in rat posterior parietal cortex. J Neurophysiol 82:2503–2517. 10561422 10.1152/jn.1999.82.5.2503

[B48] Niell CM, Stryker MP (2010) Modulation of visual responses by behavioral state in mouse visual cortex. Neuron 65:472–479. 10.1016/j.neuron.2010.01.033 20188652 PMC3184003

[B49] Nitz DA (2006) Tracking route progression in the posterior parietal cortex. Neuron 49:747–756. 10.1016/j.neuron.2006.01.037 16504949

[B50] Pachitariu M, Stringer C, Dipoppa M, Schröder S, Rossi LF, Dalgleish H, Carandini M, Harris KD (2017) Suite2p: beyond 10,000 neurons with standard two-photon microscopy. bioRxiv. Advance online publication. Retrieved May 11, 2023. doi:10.1101/061507.

[B51] Pakan JM, Lowe SC, Dylda E, Keemink SW, Currie SP, Coutts CA, Rochefort NL (2016) Behavioral-state modulation of inhibition is context-dependent and cell type specific in mouse visual cortex. Elife 5:e14985. 10.7554/eLife.14985 27552056 PMC5030095

[B52] Panzeri S, Moroni M, Safaai H, Harvey CD (2022) The structures and functions of correlations in neural population codes. Nat Rev Neurosci 23:551–567. 10.1038/s41583-022-00606-4 35732917

[B53] Park IM, Meister MLR, Huk AC, Pillow JW (2014) Encoding and decoding in parietal cortex during sensorimotor decision-making. Nat Neurosci 17:1395–1403. 10.1038/nn.3800 25174005 PMC4176983

[B54] Pfeffer CK, Xue M, He M, Huang ZJ, Scanziani M (2013) Inhibition of inhibition in visual cortex: the logic of connections between molecularly distinct interneurons. Nat Neurosci 16:1068–1076. 10.1038/nn.3446 23817549 PMC3729586

[B55] Pho GN, Goard MJ, Woodson J, Crawford B, Sur M (2018) Task-dependent representations of stimulus and choice in mouse parietal cortex. Nat Commun 9:2596. 10.1038/s41467-018-05012-y 29968709 PMC6030204

[B56] Pi H-J, Hangya B, Kvitsiani D, Sanders JI, Huang ZJ, Kepecs A (2013) Cortical interneurons that specialize in disinhibitory control. Nature 503:521–524. 10.1038/nature12676 24097352 PMC4017628

[B57] Pillow JW, Shlens J, Paninski L, Sher A, Litke AM, Chichilnisky EJ, Simoncelli EP (2008) Spatio-temporal correlations and visual signalling in a complete neuronal population. Nature 454:995–999. 10.1038/nature07140 18650810 PMC2684455

[B58] Pinto L, Goard MJ, Estandian D, Xu M, Kwan AC, Lee S-H, Harrison TC, Feng G, Dan Y (2013) Fast modulation of visual perception by basal forebrain cholinergic neurons. Nat Neurosci 16:1857–1863. 10.1038/nn.3552 24162654 PMC4201942

[B59] Polack P-O, Friedman J, Golshani P (2013) Cellular mechanisms of brain state–dependent gain modulation in visual cortex. Nat Neurosci 16:1331–1339. 10.1038/nn.3464 23872595 PMC3786578

[B60] Poulet JFA, Petersen CCH (2008) Internal brain state regulates membrane potential synchrony in barrel cortex of behaving mice. Nature 454:881–885. 10.1038/nature07150 18633351

[B61] Rikhye RV, Yildirim M, Hu M, Breton-Provencher V, Sur M (2021) Reliable sensory processing in mouse visual cortex through cooperative interactions between somatostatin and parvalbumin interneurons. J Neurosci 41:8761–8778. 10.1523/JNEUROSCI.3176-20.2021 34493543 PMC8528503

[B62] Runyan CA, Piasini E, Panzeri S, Harvey CD (2017) Distinct timescales of population coding across cortex. Nature 548:92–96. 10.1038/nature23020 28723889 PMC5859334

[B63] Saderi D, Schwartz ZP, Heller CR, Pennington JR, David SV (2021) Dissociation of task engagement and arousal effects in auditory cortex and midbrain. Elife 10:e60153. 10.7554/eLife.60153 33570493 PMC7909948

[B64] Schneider DM, Nelson A, Mooney R (2014) A synaptic and circuit basis for corollary discharge in the auditory cortex. Nature 513:189–194. 10.1038/nature13724 25162524 PMC4248668

[B65] Shimaoka D, Harris KD, Carandini M (2018) Effects of arousal on mouse sensory cortex depend on modality. Cell Rep 25:3230. 10.1016/j.celrep.2018.11.105 30540954 PMC6302665

[B66] Stitt I, Zhou ZC, Radtke-Schuller S, Fröhlich F (2018) Arousal dependent modulation of thalamo-cortical functional interaction. Nat Commun 9:2455. 10.1038/s41467-018-04785-6 29941957 PMC6018110

[B67] Stringer C, Pachitariu M, Steinmetz N, Reddy CB, Carandini M, Harris KD (2019) Spontaneous behaviors drive multidimensional, brainwide activity. Science 364:eaav7893. 10.1126/science.aav7893 PMC6525101 31000656

[B68] Taniguchi H, He M, Wu P, Kim S, Paik R, Sugino K, Kvitsiani D, Kvitsani D, Fu Y, Lu J, Lin Y, Miyoshi G, Shima Y, Fishell G, Nelson SB, Huang ZJ (2011) A resource of Cre driver lines for genetic targeting of GABAergic neurons in cerebral cortex. Neuron 71:995–1013. 10.1016/j.neuron.2011.07.026 21943598 PMC3779648

[B69] Tremblay R, Lee S, Rudy B (2016) GABAergic Interneurons in the neocortex: from cellular properties to circuits. Neuron 91:260–292. 10.1016/j.neuron.2016.06.033 27477017 PMC4980915

[B70] Tseng S-Y, Chettih SN, Arlt C, Barroso-Luque R, Harvey CD (2022) Shared and specialized coding across posterior cortical areas for dynamic navigation decisions. Neuron 110:2484–2502.e16. 10.1016/j.neuron.2022.05.012 35679861 PMC9357051

[B71] Valente M, Pica G, Bondanelli G, Moroni M, Runyan CA, Morcos AS, Harvey CD, Panzeri S (2021) Correlations enhance the behavioral readout of neural population activity in association cortex. Nat Neurosci 24:975–986. 10.1038/s41593-021-00845-1 33986549 PMC8559600

[B72] Veit J, Hakim R, Jadi MP, Sejnowski TJ, Adesnik H (2017) Cortical gamma band synchronization through somatostatin interneurons. Nat Neurosci 20:951–959. 10.1038/nn.4562 28481348 PMC5511041

[B73] Vinck M, Batista-Brito R, Knoblich U, Cardin JA (2015) Arousal and locomotion make distinct contributions to cortical activity patterns and visual encoding. Neuron 86:740–754. 10.1016/j.neuron.2015.03.028 25892300 PMC4425590

[B74] Wang X-J, Yang GR (2018) A disinhibitory circuit motif and flexible information routing in the brain. Curr Opin Neurobiol 49:75–83. 10.1016/j.conb.2018.01.002 29414069 PMC6599531

[B75] Waschke L, Tune S, Obleser J (2019) Local cortical desynchronization and pupil-linked arousal differentially shape brain states for optimal sensory performance. Elife 8:e51501. 10.7554/eLife.51501 31820732 PMC6946578

[B76] Whitlock JR, Pfuhl G, Dagslott N, Moser M-B, Moser EI (2012) Functional split between parietal and entorhinal cortices in the rat. Neuron 73:789–802. 10.1016/j.neuron.2011.12.028 22365551

[B77] Wilson NR, Runyan CA, Wang FL, Sur M (2012) Division and subtraction by distinct cortical inhibitory networks in vivo. Nature 488:343–348. 22878717 10.1038/nature11347 PMC3653570

[B78] Xiang Z, Huguenard JR, Prince DA (1998) Cholinergic switching within neocortical inhibitory networks. Science 281:985–988. 10.1126/science.281.5379.985 9703513

[B79] Xu H, Jeong H-Y, Tremblay R, Rudy B (2013) Neocortical somatostatin-expressing GABAergic interneurons disinhibit the thalamorecipient layer 4. Neuron 77:155–167. 10.1016/j.neuron.2012.11.004 23312523 PMC3556168

[B80] Yavorska I, Wehr M (2021) Effects of locomotion in auditory cortex are not mediated by the VIP network. Front Neural Circuits 15:618881. 10.3389/fncir.2021.618881 33897378 PMC8058405

[B81] Zhou M, Liang F, Xiong XR, Li L, Li H, Xiao Z, Tao HW, Zhang LI (2014) Scaling down of balanced excitation and inhibition by active behavioral states in auditory cortex. Nat Neurosci 17:841–850. 10.1038/nn.3701 24747575 PMC4108079

