# Supplementary figures and images for: Arousal and Locomotion Differently Modulate Activity of Somatostatin Neurons across Cortex
Source: eNeuro. 2023 May 24;10(5):ENEURO.0136-23.2023. doi: 10.1523/ENEURO.0136-23.2023 (PMC10216262; doi:10.1523/ENEURO.0136-23.2023)

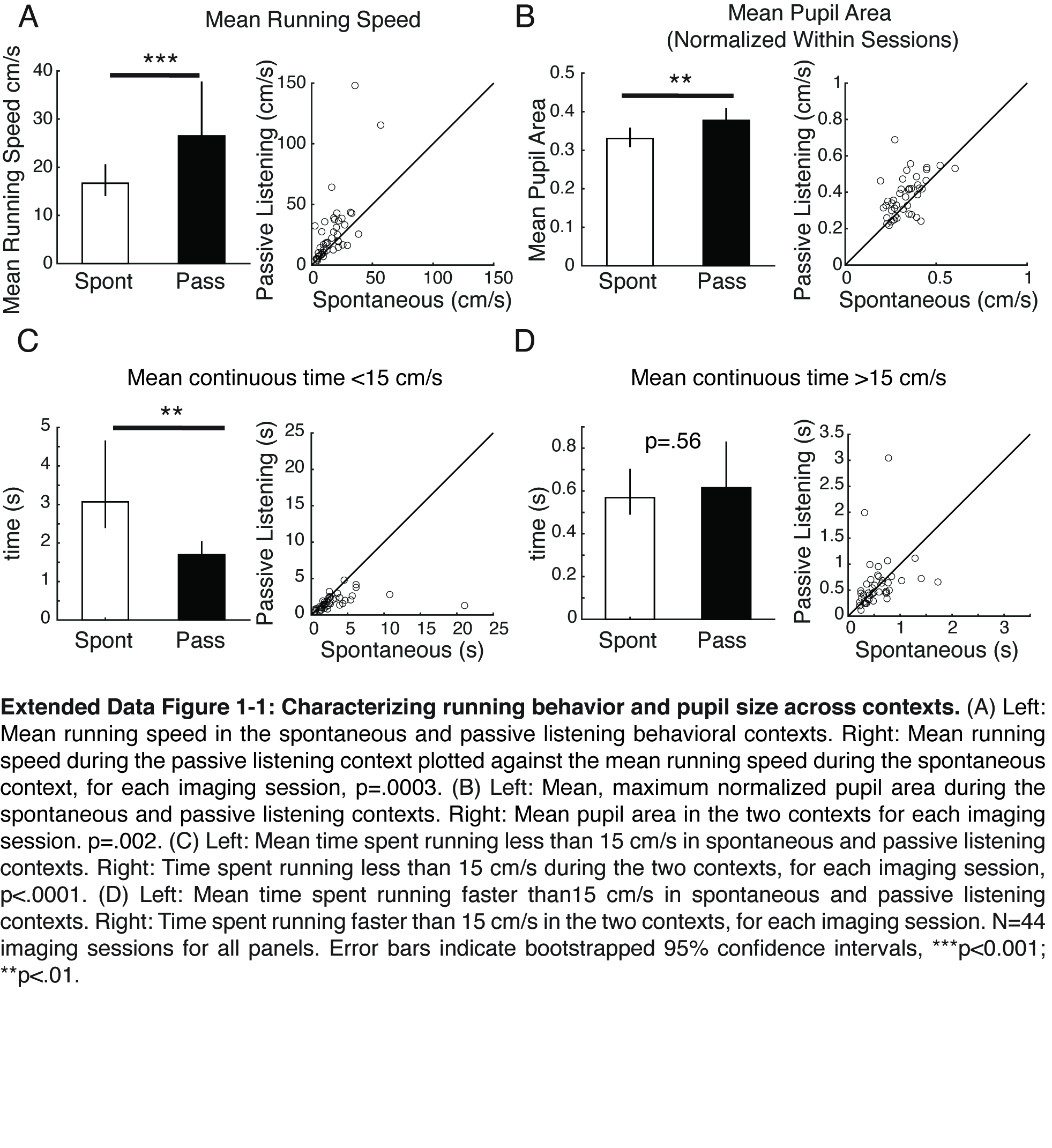

Supplement: Figure 1-1 — Characterizing running behavior and pupil size across contexts. A, Left, Mean running speed in the spontaneous and passive listening behavioral contexts. Right, Mean running speed during the passive listening context plotted against the mean running speed during the spontaneous context, for each imaging session, p = 0.0003. B, Left, Mean, maximum normalized pupil area during the spontaneous and passive listening contexts. Right, Mean pupil area in the two contexts for each imaging session, p = 0.002. C, Left, Mean time spent running <15 cm/s in spontaneous and passive listening contexts. Right, Time spent running <15 cm/s during the two contexts, for each imaging session, p < 0.0001. D, Left, Mean time spent running >15 cm/s in spontaneous and passive listening contexts. Right, Time spent running >15 cm/s in the two contexts, for each imaging session. N = 44 imaging sessions for all panels. Error bars indicate bootstrapped 95% confidence intervals. ***p < 0.001; **p < 0.01. Download Figure 1-1, TIF file. [file enu-eN-NWR-0136-23-s02.tif]

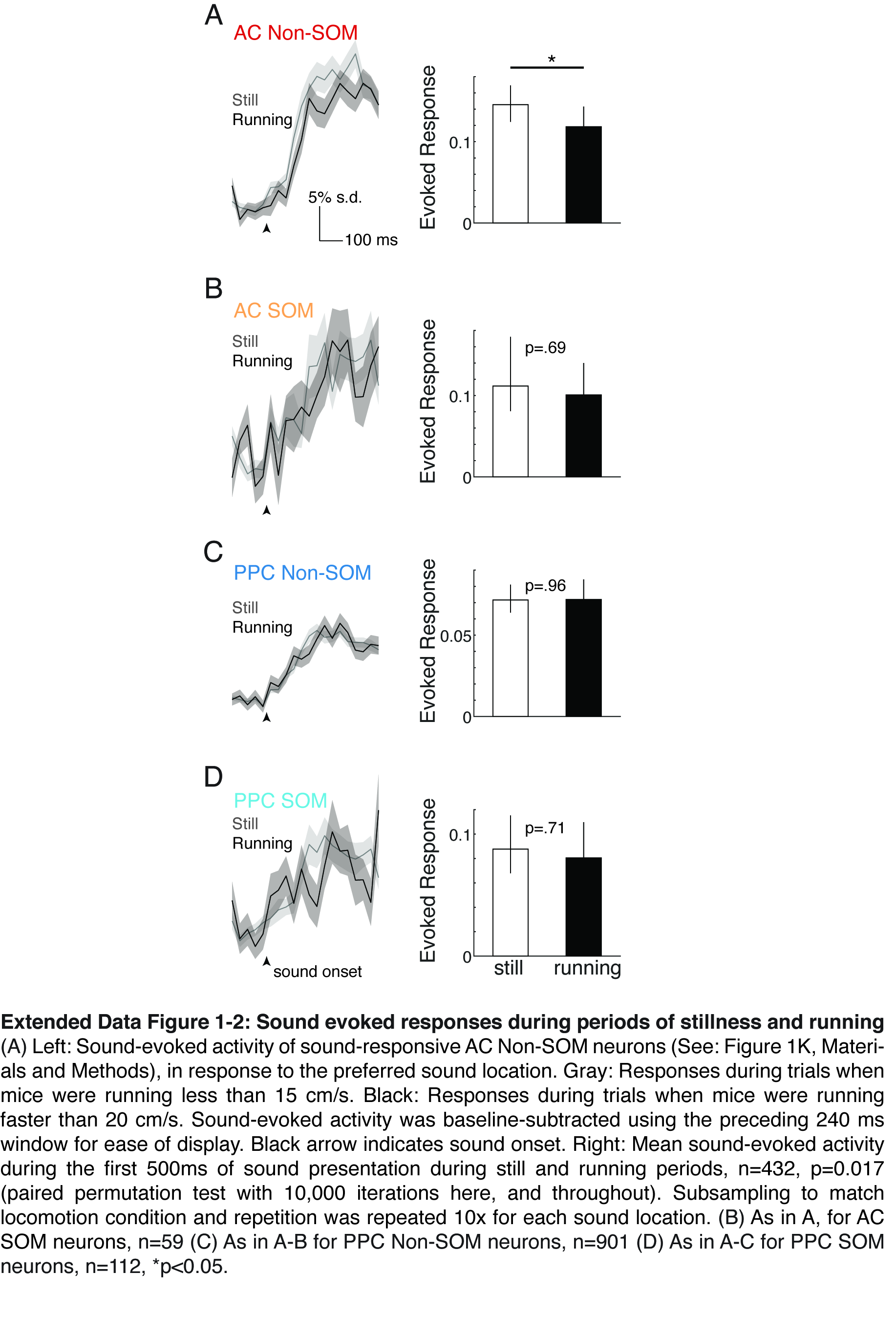

Supplement: Figure 1-2 — Sound-evoked responses during periods of stillness and running. A, Left, Sound-evoked activity of sound-responsive AC Non-SOM neurons (Fig. 1K; Materials and Methods), in response to the preferred sound location. Gray, Responses during trials when mice were running <15 cm/s; black, responses during trials when mice were running >20 cm/s. Sound-evoked activity was baseline subtracted using the preceding 240 ms window for ease of display. Black arrow indicates sound onset. Right, Mean sound-evoked activity during the first 500 ms of sound presentation during still and running periods, n = 432, p = 0.017 (paired permutation test with 10,000 iterations here, and throughout). Subsampling to match locomotion condition and repetition was repeated 10 times for each sound location. B, As in A, for AC SOM neurons, n = 59. C, As in A and B for PPC Non-SOM neurons, n = 901. D, As in A–C for PPC SOM neurons, n = 112. *p < 0.05. Download Figure 1-2, TIF file. [file enu-eN-NWR-0136-23-s01.tif]
